# The cytoskeletal protein profilin is an important allergen in saltwort (*Salsola kali*)

**DOI:** 10.3389/fimmu.2024.1379833

**Published:** 2024-06-07

**Authors:** Ludmila Peterkova, Daria Trifonova, Pia Gattinger, Margarete Focke-Tejkl, Victoria Garib, Nigora Magbulova, Gulnara Djambekova, Nodira Zakhidova, Mokhigul Ismatova, Bulent Enis Sekerel, Sevda Tuten Dal, Mikhail Tulaev, Michael Kundi, Walter Keller, Alexander Karaulov, Rudolf Valenta

**Affiliations:** ^1^Institute of Pathophysiology and Allergy Research, Center for Pathophysiology, Infectiology and Immunology, Medical University of Vienna, Vienna, Austria; ^2^Laboratory of Immunopathology, Department of Clinical Immunology and Allergy, Sechenov First Moscow State Medical University, Moscow, Russia; ^3^Karl Landsteiner University, Krems an der Donau, Austria; ^4^Ministry of Higher Education, Science and Innovation, Tashkent, Uzbekistan; ^5^International Center of Molecular Allergology, Ministry of Higher Education, Science and Innovation, Tashkent, Uzbekistan; ^6^International Allergy Center, Tashkent, Uzbekistan; ^7^Pediatric Allergy and Asthma Division, Hacettepe University School of Medicine, Ankara, Türkiye; ^8^Department of Environmental Health, Center for Public Health, Medical University of Vienna, Vienna, Austria; ^9^Institute of Molecular Biosciences, BioTechMed Graz, University of Graz, Graz, Austria; ^10^National Research Center, National Research Center Institute of Immunology (NRCI) Institute of Immunology, Federal Medical-Biological Agency of Russia (FMBA), Moscow, Russia

**Keywords:** allergy, allergen molecules, profilin, pollen allergy, *Salsola kali*, diagnosis, allergen-specific immunotherapy

## Abstract

Pollen from *Salsola kali*, i.e., saltwort, Russian thistle, is a major allergen source in the coastal regions of southern Europe, in Turkey, Central Asia, and Iran. *S. kali-*allergic patients mainly suffer from hay-fever (i.e., rhinitis and conjunctivitis), asthma, and allergic skin symptoms. The aim of this study was to investigate the importance of individual *S. kali* allergen molecules. Sal k 1, Sal k 2, Sal k 3, Sal k 4, Sal k 5, and Sal k 6 were expressed in *Escherichia coli* as recombinant proteins containing a C-terminal hexahistidine tag and purified by nickel affinity chromatography. The purity of the recombinant allergens was analyzed by SDS-PAGE. Their molecular weight was determined by matrix-assisted laser desorption/ionization time-of-flight mass spectrometry, and their fold and secondary structure were studied by circular dichroism (CD) spectroscopy. Sera from clinically well-characterized *S. kali*-allergic patients were used for IgE reactivity and basophil activation experiments. *S. kali* allergen-specific IgE levels and IgE levels specific for the highly IgE cross-reactive profilin and the calcium-binding allergen from timothy grass pollen, Phl p 12 and Phl p 7, respectively, were measured by ImmunoCAP. The allergenic activity of natural *S. kali* pollen allergens was studied in basophil activation experiments. Recombinant *S. kali* allergens were folded when studied by CD analysis. The sum of recombinant allergen-specific IgE levels and allergen-extract-specific IgE levels was highly correlated. Sal k 1 and profilin, reactive with IgE from 64% and 49% of patients, respectively, were the most important allergens, whereas the other *S. kali* allergens were less frequently recognized. Specific IgE levels were highest for profilin. Of note, 37% of patients who were negative for Sal k 1 showed IgE reactivity to Phl p 12, emphasizing the importance of the ubiquitous cytoskeletal actin-binding protein, profilin, for the diagnosis of IgE sensitization in *S. kali*-allergic patients. rPhl p 12 and rSal k 4 showed equivalent IgE reactivity, and the clinical importance of profilin was underlined by the fact that profilin-monosensitized patients suffered from symptoms of respiratory allergy to saltwort. Accordingly, profilin should be included in the panel of allergen molecules for diagnosis and in molecular allergy vaccines for the treatment and prevention of *S. kali* allergy.

## 1 Introduction

Pollen from weeds, in particular from the plant families of *Asteraceae, Amaranthaceae, Plantaginaceae, Urticaceae*, and *Euphorbiaceae*, represents an important allergen source worldwide (1). In particular, the family of *Amaranthaceae* comprising three major genera, *Chenopodium, Amaranthus*, and *Salsola*, is considered to be highly relevant in Middle Asia, southern Europe, western United States, Saudi Arabia, Kuwait, and Iran (2–5). Russian thistle, also termed saltwort (*Salsola kali*), is a widely distributed weed and was discovered as an important allergenic plant in the United States already in 1933 (6). *S. kali*-sensitized allergic patients mainly suffer from allergic rhinoconjunctivitis, dermatitis, and severe and disabling asthma (7). Initial attempts for the characterization of *S. kali* allergens focused on the protein chemical characterization of the allergenic components (8). With the application of molecular biological methods for allergen characterization, our knowledge regarding the individual allergen molecules in different allergen sources, including *S. kali*, has rapidly increased (2, 9, 10).

According to the WHO/IUIS allergen nomenclature database^1^, the primary structures of seven allergens from *S. kali* are currently described. These seven allergens comprise a pectin methylesterase Sal k 1, a protein kinase homologue Sal k 2, a cobalamin-independent methionine synthase Sal k 3, a profilin Sal k 4, an Ole e 1-like protein Sal k 5, a polygalacturonase Sal k 6, and a polcalcin Sal k 7. The major allergen Sal k 1, a pectin methylesterase, is a ubiquitous cell wall-associated enzyme occurring in several isoforms, facilitating plant cell wall modification and subsequent breakdown. Pectin esterases are glycoproteins affecting mechanical stability of cell walls, for example, in the process of fruit ripening. They also contribute to cell wall extension during pollen germination and pollen tube growth, and are involved in tuber yield and root development. In addition, pectin esterases are considered as proteins defending plants against attack of pathogens (11, 12). Sal k 2 is a protein kinase homologue with a molecular mass of approximately 36 kDa, possessing a conserved catalytic core (1). Cobalamin-independent methionine synthase from *S. kali*, termed Sal k 3, is a rather large protein with a molecular mass of approximately 85 kDa (13, 14). Sal k 4 was identified as the cytoskeletal protein profilin in saltwort pollen (15, 16). Profilin is an actin-binding protein involved in the acrosomal reaction, which has been known for a long while in eukaryotic organisms and was discovered in plants initially as a highly cross-reactive allergen by screening of a birch pollen expression cDNA library with allergic patients IgE (17, 18). Shortly thereafter, it was established as an actin-binding protein also in higher plants (19). Sal k 5 belongs to the Ole e 1-like protein family (20). Sal k 5 is a glycoprotein with a molecular weight of approximately 17 kDa, which has been reported to be recognized by IgE from up to 40% of saltwort pollen allergic patients (20). Sal k 5 shares a high degree of amino acid sequence identity (i.e., 68%) with Che a 1, the major allergen from *Chenopodium album* pollen, and there is extensive IgE cross-reactivity between the two allergens. Conversely, little or no IgE cross-reactivity between Sal k 5 and the major olive tree pollen allergen Ole e 1 has been reported (20, 21). Sal k 6, a polygalacturonase, occurs in Russian thistle pollen as a 47-kDa glycoprotein. It represents an enzyme that hydrolyzes the α-1,4 glycosidic bonds between galacturonic acid residues and thus has an effect on cell walls, like Sal k 1 has. The protein is similar to other allergenic polygalacturonases from *Platanaceae, Poaceae*, and *Cupressaceae*, but the sequence similarity with these allergens is very low (i.e., 20%–45% sequence identity), and accordingly, there seems to be little if any IgE cross-reactivity (22). Sal k 6 has been described as a minor allergen, being recognized by approximately 30% of saltwort-allergic patients. Sal k 7 is a two EF-hand calcium binding allergen that represents a highly cross-reactive plant pollen allergen (23–27). The *S. kali* allergens have been described individually by different authors and are listed in the allergen nomenclature database^2^ and in certain review articles (1, 2, 28), yet they have not been compared regarding their IgE binding and their association with clinical symptoms in clinically well-characterized patients with *S. kali*-related allergic symptoms. Accordingly, there is a need to study the role of the individual saltwort allergens for saltwort allergy. The frequency of IgE recognition is only one parameter to establish the relevance of an allergen whereas further parameters need to be assessed such as the clinical relevance (29, 30). The determination of the clinical relevance can be investigated by provocation testing, by studying the allergenic activity but most importantly, by identifying patients with clinical symptoms who are monosensitized against a certain allergen.

The aim of this study was to express and purify a comprehensive panel of saltwort allergen molecules Sal k 1, 2, 3, 4, 5, and 6 in non-glycosylated form to avoid *S. kali*-unrelated IgE reactivities to cross-reactive carbohydrate determinants (CCDs) (31). The highly cross-reactive timothy grass polcalcin, Phl p 7 (polcalcin), and profilin, Phl p 12, were included in the analysis to represent the immunologically equivalent *Salsola* proteins, Sal k 4 and Sal k 7, respectively. Allergen-specific IgE levels to the individual allergen molecules were quantified by ImmunoCAP technology and related to clinical symptoms. In addition, we studied the association of IgE sensitization to the individual saltwort allergens with clinical symptoms. Our study confirms that Sal k 1 is the most frequently recognized saltwort allergen but, surprisingly, profilin turned out to be the second most frequently recognized allergen, which accounted for high levels of *S. kali*-specific IgE in the patients. The clinical importance of Sal k 4 is highlighted by the fact that Sal k 4-monosensitized patients suffered from allergic symptoms to saltwort. These findings are relevant for molecular diagnosis and allergen-specific immunotherapy of *S. kali* allergy.

## 2 Results

### 2.1 Demographic and clinical characterization of *Salsola kali*-allergic patients

**Figure 1:**
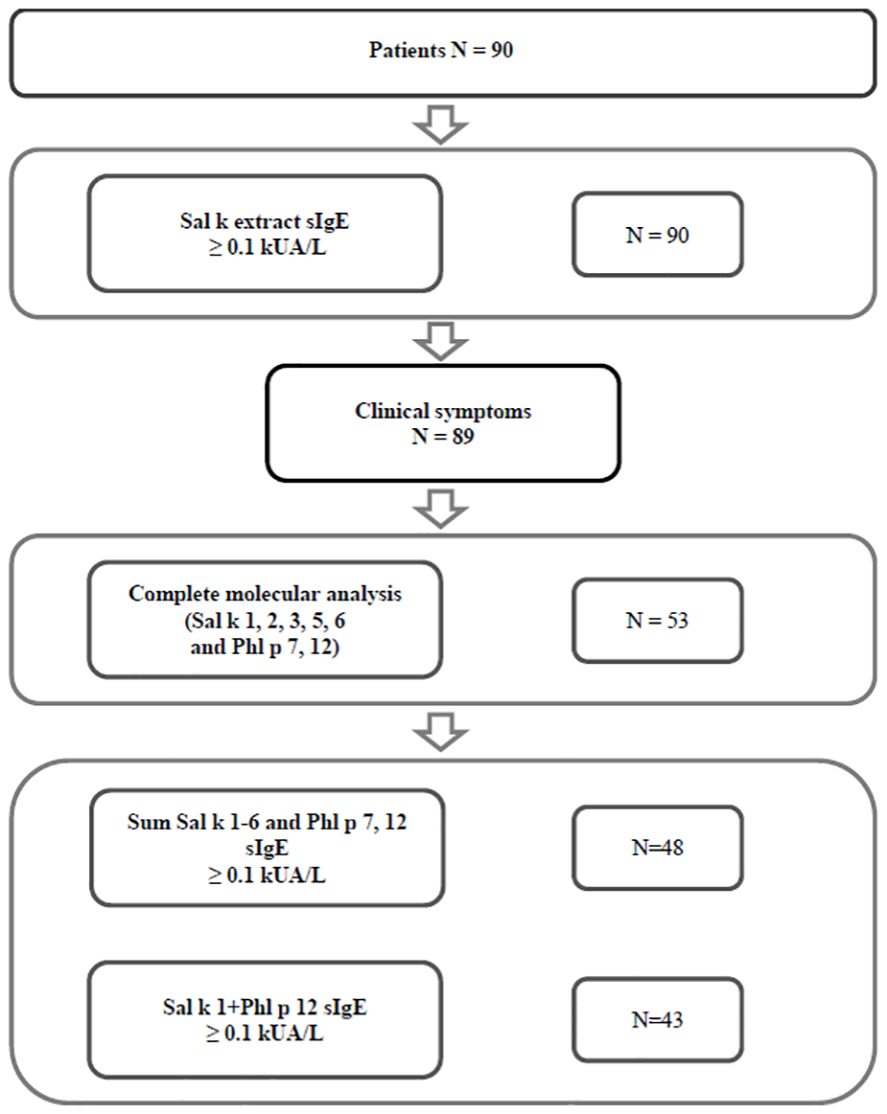
Consort diagram of patients, analyses, and results.

Sera from *S. kali* sensitized patients from two regions with high exposure to *S. kali* pollen were obtained, i.e., Tashkent region (Uzbekistan) (*n* = 77) and the Republic of Türkiye (*n* = 13) (**Figure 1**). A detailed demographic, clinical, and serological description of the patients can be found in **Table 1** and **Supplementary Table 1**. The consort diagram in **Figure 1** shows the enrolment of patients for this study and the analyses that were performed. In a first step, patients were tested for specific IgE antibodies, and it was found that all of them (*n* = 90) had Sal k allergen extract-specific IgE ≥ 0.1 kU_A_/L (**Supplementary Table 1**). All but one of these patients had clinical symptoms upon exposure to *S. kali* pollen (**Figure 1**, **Table 1**, **Supplementary Table 1**). Available volumes of sera allowed the analysis of 53 sera for IgE reactivity to all allergens, i.e., Sal k 1, 2, 3, 5, and 6, the cross-reactive pollen allergens Phl p 7 and Phl p 12, and the carbohydrate marker HRP (**Figure 1**, **Table 1**, **Supplementary Table 1**).

**Table 1:** Demographic and clinical characteristics of patients with clinical symptoms of saltwort pollen allergy, and of symptomatic patients with saltwort allergen-specific IgE antibodies tested with all components.

Sex		Age Mean (Min–Max)	Symptoms upon saltwort pollen exposure			
M	F		Asthma	Rhinitis	Conjunctivitis	Dermatitis
49	40	24.30 (5–62)	28	88	52	10
Patients with specific IgE antibodies ≥ 0.1 kU_A_/L tested with all components (*N* = 53)						
30	23	22.98 (6–62)	14	52	32	6

Out of the 89 *S. kali*-symptomatic patients, 49 were male and 40 female patients with a mean age of 24.30 years (**Table 1**). The most common symptoms were rhinitis (*n* = 88) > conjunctivitis (*n* = 52) > asthma (*n* = 28) and dermatitis (*n* = 10) (**Table 1**, **Supplementary Table 1**). A total of 53 patients tested for all allergen molecules comprised 30 male and 23 female patients with a mean age of 22.98 years. Regarding clinical symptoms, the latter patients were representative of the whole group (rhinitis: *n* = 52 > conjunctivitis: *n* = 32 > asthma: *n* = 14 and dermatitis: *n* = 6) (**Table 1**, **Supplementary Table 1**). In **Supplementary Table 1**, patients were identified who had one to two, three, or four symptoms to *S. kali* pollen.

### 2.2 Characterization of recombinant *Salsola kali* allergen molecules and of recombinant *Phleum pratense* profilin

Sal k 1, Sal k 2, Sal k 3, Sal k 4, Sal k 5, and Sal k 6 were expressed as recombinant His-tagged proteins in *Escherichia coli* and could be purified as soluble proteins. Recombinant proteins were analyzed under reducing conditions by SDS-PAGE (**Supplementary Figure 1**), by immunoblotting with anti-His antibodies (**Supplementary Figure 2**), and by mass spectrometry (**Supplementary Figure 3**). The molecular mass calculated for Sal k 1 with a, N-terminal methionine was 37,770 Da, which corresponded to the one determined by mass spectrometry (i.e., 37,798 Da) and which was in agreement with the migration of the protein in SDS-PAGE and by immunoblotting with anti-His antibodies (i.e., approximately 37 kDa) (**Supplementary Figures 1–3**). The molecular weight deduced from the sequence of Sal k 2 is 36,655 Da without N-terminal methionine, which corresponds to a broad peak determined by mass spectrometry in the range of 35,600 Da and a band migrating in SDS-PAGE at approximately 36 kDa, which reacted with anti-His antibodies (**Supplementary Figures 1–3**). For Sal k 3, the molecular mass calculated according to the sequence including the N-terminal methionine was 84,385 Da which is in good agreement with the molecular mass determined by mass spectrometry (i.e., 85,388 Da) and a band of approximately 85 kDa found in SDS-PAGE, which reacted with anti-His antibodies (**Supplementary Figures 1–3**). A light band, likely representing a degradation product at approximately 30 kDa, was observed by SDS-PAGE and reacted with anti-His antibodies (**Supplementary Figures 1, 2**).

The molecular mass predicted for Sal k 4 with N-terminal methionine was 15,095 Da, which corresponded to the molecular mass determined by mass spectrometry (i.e., 15,256 Da) (**Supplementary Figures 1–3**). The band at approximately 15 kDa observed for Sal k 4 in SDS-PAGE and by immunoblotting with the anti-His antibodies was in agreement with the aforementioned data. The molecular mass calculated according to the amino acid sequence of Sal k 5 including the N-terminal methionine (i.e., 17,402 Da) corresponded to the mass determined by mass spectrometry (i.e., 17,433 Da) and was in agreement with the migration of rSal k 5 in SDS-PAGE (**Supplementary Figures 1, 3**). rSal k 5 migrated in SDS-PAGE and was detected by anti-His immunoblotting at approximately 17 kDa (**Supplementary Figures 1, 2**). An additional band at approximately 35 kDa, probably representing a dimer, was observed in SDS-PAGE and by anti-His immunoblotting corresponding to an additional peak observed by mass spectrometry at 33,700 Da (**Supplementary Figures 1–3**). For Sal k 6, it has been reported that the allergen undergoes natural degradation (22). We calculated according to the amino acid sequence of Sal k 6 a molecular mass of 42,757 Da without and 42,889 Da with methionine at the N-terminus. A band of this size was also observed by SDS-PAGE and reacted with anti-His antibodies by immunoblotting (**Supplementary Figures 1, 2**). Additional smaller bands were observed by SDS-PAGE, which reacted with anti-His antibodies at 28 kDa and 20 kDa, corresponding to two peaks observed by mass spectrometry at 27,053 Da and 22,961 Da (**Supplementary Figures 1–3**). The bands observed by SDS-PAGE at approximately 12 kDa and 13 kDa corresponded to peaks at 12,382 Da and 13,730 Da observed by mass spectrometry, respectively (**Supplementary Figures 1, 3**). By mass spectrometry, additional peaks at approximately 16,000 Da, 8,360 Da, and 7,485 Da were observed. The latter two peaks sum up with the peak at 35,403 Da to yield the predicted mass of 42,757 Da to 42,889 Da, suggesting that the bands observed by SDS-PAGE and immunoblotting may have undergone fragmentation during mass spectrometry.

rPhl p 12 was expressed in *E. coli* and purified as folded recombinant His-tagged protein as previously described (32).

Next, we performed an analysis of the fold of the recombinant *S. kali* allergens by circular dichroism (CD) analysis (**Supplementary Figure 4**). rSal k 1 showed a CD spectrum with a broad minimum at 216 nm, indicating a high β-sheet secondary structure content. This corresponds well with the alphaFold2 prediction, which yields a right-handed parallel β-helix fold and a structural homology to the family of pectin methylesterases. rSal k 2 exhibited a CD spectrum with a pronounced minimum at 208 nm and a broad shoulder at 222 nm, indicating a fold of mainly α-helical secondary structure. This is consistent with the AlphaFold2 prediction of a protein kinase-like fold. It should be mentioned in this respect that the predicted model features a long N-terminal α-helix with low per-residue model confidence score (pLDDT) values for the first 55 amino acids and therefore this part of the protein may well be unstructured in solution. This α-helix, however, is not part of the protein kinase-like fold and does not affect the kinase-like fold itself, which exhibits a high model confidence (pLDDT > 70). rSal k 3, which was proposed to be a cobalamin-independent methionine synthase, exhibits in its CD spectrum a minimum at 208 nm and a broad shoulder with an indicated smaller minimum at 222 nm. This is in good agreement with the AlphaFold2 prediction of the protein, which shows a two-domain structure with a mixed α/β-fold. The CD spectrum showed that rSal k 4 was a folded protein with a mixed α-helical, β-sheet fold almost identical to Phl p 12 (32).

Sal k 5 is an Ole e 1-like protein and the AlphaFold2 prediction exhibits a seven-stranded anti-parallel β-barrel with an adjacent α-helix and a small α-helix interrupting one of the β-strands. This corresponds well with the measured CD spectrum, exhibiting a minimum at 208 nm and a broad shoulder at longer wavelengths. Sal k 6 has been identified as a polygalacturonase enzyme. The CD spectrum exhibits a broad, shallow minimum extending from 208 to 220 nm, consistent with the AlphaFold2 prediction of a right-handed parallel β-helix.

### 2.3 Serological characterization of patients with *Salsola kali* allergy

**Figure 1** shows that 89 out of 90 patients who reported symptoms to *S. kali* had IgE antibody levels ≥ 0.1 kU_A_/L to *S. kali* pollen allergen extract by ImmunoCAP measurements (**Figure 1**, **Supplementary Table 1**). Regarding the 53 patients who could be tested for IgE reactivity to recombinant Sal k 1, 2, 3, 5, and 6, and Phl p 7 and 12, we found that 90.57% had specific IgE antibodies ≥ 0.1 kU_A_/L to the sum of Sal k 1–6 and Phl p 7 and Phl p 12 (**Figure 1**, **Supplementary Table 1**) and 81% showed IgE reactivity to the combination of rSal k 1 and rPhl p 12 (**Figure 1**, **Supplementary Table 1**).

### 2.4 rSal k 1 is the major *Salsola kali* allergen but profilin is the second most frequently recognized allergen accounting for the highest specific IgE levels

Next, we quantified allergen-specific IgE levels to the individual *S. kali* allergens, Sal k 1–6, and the cross-reactive pollen allergens Phl p 7 and Phl p 12 (33, 34).

Raw data for the allergen-specific IgE levels for all 89 patients with saltwort-related symptoms are outlined in **Supplementary Table 1**. **Table 2** summarizes the percentages of patients reactive for each allergen molecule and median values of allergen-specific IgE levels for those of the 53 patients who were positive (i.e., ≥ 0.1 kU_A_/L) for each of the allergen molecules. The frequencies of IgE recognition for the allergen molecules were 64.15% (Sal k 1) > 49.06% (Phl p 12) > 22.64% (Sal k 5, Sal k 6) > 13.21% (Sal k 2) > 11.32% (Phl p 7) > 9.43% (Sal k 3). Phl p 12 was used for testing instead of *S. kali* profilin, Sal k 4, because it was found to cross-react with Sal k 4 as determined with rabbit anti-Phl p 12 antibodies and by IgE inhibition studies and thus is immunologically equivalent (**Supplementary Figure 4**). IgE binding to Sal k 4 could be inhibited completely with rPhl p 12 (**Supplementary Figure 5**). Thus, we confirm that Sal k 1 represents the major *S. kali* allergen regarding the frequency of IgE recognition. Sal k 1 occurs in the form of almost identical isoforms (**Supplementary Figures 6**) and belongs to the family of pectin esterases (**Supplementary Figure 6**). In fact, it shows very close sequence relationship to pectin esterases from beetroot, spinach, and *Amaranthus* (**Supplementary Figure 6**).

**Table 2:** Serological characteristics of positive symptomatic patients with *Salsola kali* allergen-specific IgE antibodies (≥0.1 kU_A_/L), who were tested with all components.

A										
	Sal k extract	rSal k 1	rSal k 2	rSal k 3	rSal k 5	rSal k 6	rPhl p 7	rPhl p 12	Sal k 1 + Phl p 12	Sum Sal k 1–6 and Phl p 7, 12
kU_A_/L median (min–max)	9.7	0.55	0.15	0.14	0.85	0.64	0.29	2.46	2.15	2.45
	(0.57–84.3)	(0.1–9.9)	(0.1–0.59)	(0.1–1.08)	(0.11–8.51)	(0.11–15.5)	(0.1–4.61)	(0.13–99.5)	(0.11–99.62)	(0.1–100.29)
B										
	Sal k extract	rSal k 1	rSal k 2	rSal k 3	rSal k 5	rSal k 6	rPhl p 7	rPhl p 12	Sal k 1 + Phl p 12	Sum Sal k 1–6 and Phl p 7, 12
*N* %	*N* = 53	*N* = 34	*N* = 7	*N* = 5	*N* = 12	*N* = 12	*N* = 6	*N* = 26	*N* = 43	*N* = 48
	100%	64.15%	13.21%	9.43%	22.64%	22.64%	11.32%	49.06%	81.13%	90.57%

(A) Ranges and median values of allergen-specific IgE levels. (B) Numbers and percentages of patients with allergen-specific IgE antibodies.

We compared rPhl p 12 and rSal k 4 regarding IgE reactivity using sera from 29 patients who had shown IgE reactivity to Phl p 12 by ImmunoCAP testing (**Supplementary Tables 1, 2**). **Supplementary Table 2** shows the IgE reactivity of Sal k 4 and Phl p 12 as determined by ELISA. We found fully concordant IgE reactivity of Sal k 4 and Phl p 12 (**Supplementary Table 2**, **Supplementary Figures 7A, B**). For certain patients, IgE reactivity to Sal k 4 was even higher than for Phl p 12, confirming the importance of Sal k 4 as allergen in saltwort (**Supplementary Table 2**), but there was no significant difference between IgE levels to rSal k 4 and rPhl p 12 (**Supplementary Figure 7A**) and rSal k 4 and rPhl p 12-specific IgE levels were correlated in a highly significant manner (**Supplementary Figure 7B**).

Phl p 7 was used instead of Sal k 7 because of its structural similarity with Sal k 7. In fact, the sequence homology between Sal k 4 and Phl p 12 was 93% and that between Sal k 7 and Phl p 7 was 83% (**Supplementary Figure 8**). Profilin, as represented by the cross-reactive Phl p 12, could be almost considered as a major allergen in our population because it was recognized by nearly 50% of the patients. Furthermore, the median level of Phl p 12-specific IgE (i.e., 2.46 kU_A_/L) was higher than that for any of the other tested *S. kali* allergens and accounted for almost a third of the IgE directed against natural *S. kali* allergens (**Table 2**). Importantly, we found that 7 out of the 19 patients (i.e., patient #8, 18, 39, 45, 46, 72, and 77) who were negative for Sal k 1 showed IgE reactivity to Phl p 12, emphasizing the importance of including profilin for detecting IgE sensitization to *S. kali*. All but two patients with IgE sensitization to Phl p 12 were sensitized also to at least one of the following marker allergens indicative for IgE sensitization to pollen from mugwort (Art v 1), cypress (Cry j 1), cedar (Cup a 1), olive (Ole e 1), plane tree (Pla a 1), or timothy grass (Phl p 1, Phl p 2, and Phl p 5), indicating that they are poly-sensitized to pollen from different plants (data not shown). By contrast, the equivalent of the calcium-binding allergen Sal k 7 (i.e., Phl p 7) reacted only with IgE from 11% of the *S. kali-*allergic patients (**Table 2**).

**Figure 2:**
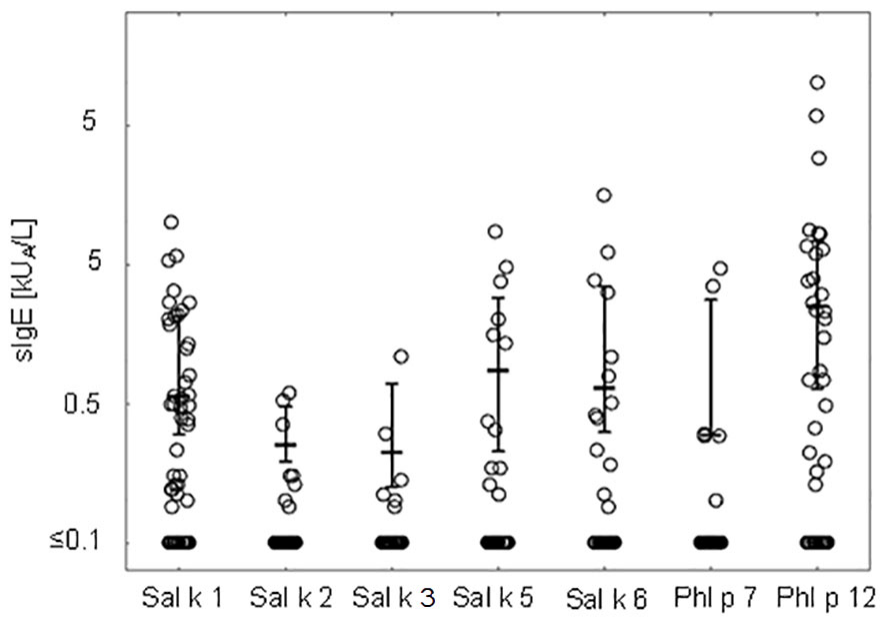
IgE levels (medians and interquartile ranges for values above the cutoff level ≥0.1 kU_A_/L are denoted) specific to *Salsola kali* and cross-reactive allergen molecules.

**Figure 2** shows the comparison of median IgE levels specific for each of the tested recombinant allergens illustrating that the median levels of Phl p 12-specific IgE are higher than those for the other allergen molecules.

**Table 3** presents pairwise comparisons of IgE levels specific for each of the tested allergen molecules as indicated in **Figure 2**. This type of pairwise analysis revealed significant differences for the allergen-specific IgE levels as indicated by *p*-values printed in bold. Although the median of the Phl p 12-specific IgE levels was higher than that for Sal k 1, no significant difference regarding the pairwise comparison of specific IgE levels to Sal k 1 and Phl p 12 was found (**Table 3**). However, Phl p 12- and Sal k 1-specific IgE levels were significantly higher than those for the other Sal k allergens in the pairwise comparisons (**Table 3**).

**Table 3:** Pairwise comparison between allergens (Tukey’s HSD test).

Allergen	Sal k 1	Sal k 2	Sal k 3	Sal k 5	Sal k 6	Phl p 7	Phl p 12
Sal k 1		**0.000026**	**0.000026**	**0.000313**	**0.000819**	**0.000026**	0.984178
Sal k 2	**0.000026**		0.999981	0.664348	0.508782	0.999917	**0.000026**
Sal k 3	**0.000026**	0.999981		0.497227	0.349697	0.997729	**0.000026**
Sal k 5	**0.000313**	0.664348	0.497227		0.999988	0.847827	**0.000030**
Sal k 6	**0.000819**	0.508782	0.349697	0.999988		0.720862	**0.000042**
Phl p 7	**0.000026**	0.999917	0.997729	0.847827	0.720862		**0.000026**
Phl p 12	0.984178	**0.000026**	**0.000026**	**0.000030**	**0.000042**	**0.000026**	

*p*-values are shown (bold: *p* < 0.05).

### 2.5 IgE specific for the sum of recombinant allergen molecules significantly correlates with allergen extract-specific IgE

**Figure 3:**
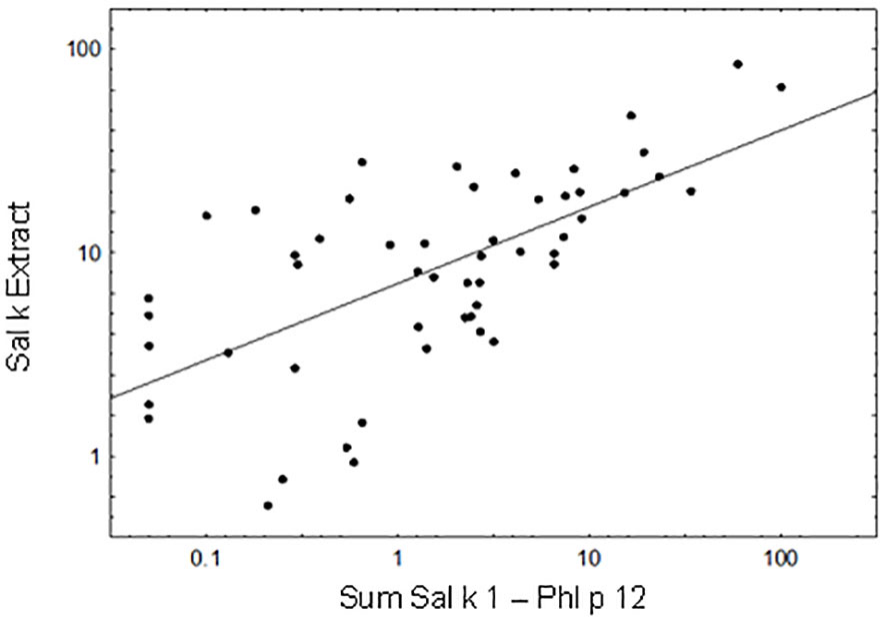
Correlation between the cumulative sum of Sal k 1-Phl p 12-specific IgE antibody levels and IgE levels to Sal k extract. Pearson’s correlation coefficient *r* = 0.646, *p* < 0.00001.

We then performed a Pearson’s correlation analysis of the cumulative IgE levels specific for the sum of Sal k 1–6 and Phl p 12 versus IgE levels specific for natural *S. kali* allergens (**Figure 3**). A significant correlation could be observed between the cumulative allergen-specific IgE levels (the cumulative sum of IgE levels specific to the allergen molecules) and IgE levels specific to saltwort extract (**Figure 3**; *p* < 0.00001, Pearson’s correlation coefficient *r* = 0.646). Only 5 out of the 53 patients (i.e., patient #2, 5, 26, 63, and 66) who were IgE-positive with the allergen extract were negative with the available allergen molecules at the given cutoff (**Supplementary Table 1**). We also observed that cumulative IgE levels of the tested allergen molecules were higher than those of the allergen extract in one of the Phl p 12-sensitized patients (i.e., patient #79) (**Supplementary Table 1**).

### 2.6 Association of symptoms with IgE recognition of allergens

**Figure 4:**
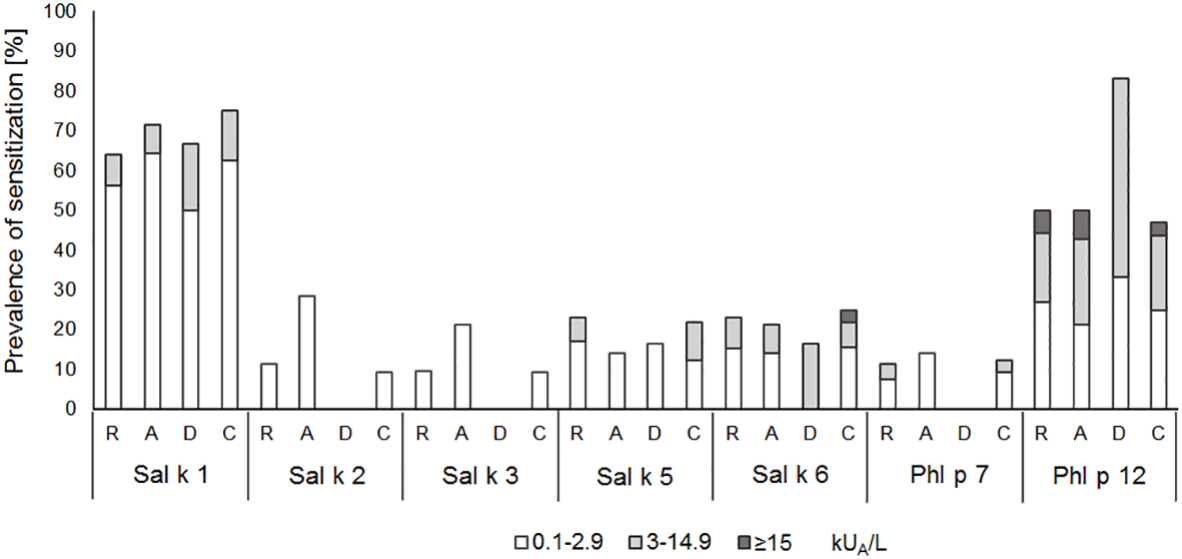
Prevalences and magnitudes of IgE sensitization to *Salsola kali*- and cross-reactive allergen molecules in *Salsola kali*-allergic patients (*n* = 53) with different symptoms (R, rhinitis; A, asthma; D, dermatitis; C, conjunctivitis).

In another set of experimental approaches, we searched for serological markers that may be associated with phenotypes of *S. kali* sensitization. First, we studied whether IgE sensitization and/or IgE levels specific for certain allergen molecules are associated with certain symptoms. **Figure 4** shows the prevalences and IgE levels specific for the recombinant allergen molecules for different types of symptoms. No relevant association of IgE sensitization to a particular allergen and certain symptoms was found. We only noted that none of the six patients with dermatitis reacted with Sal k 2, Sal k 3, and the cross-reactive Phl p 7 allergen, whereas other allergens were recognized (**Figure 4**).

**Figure 5:**
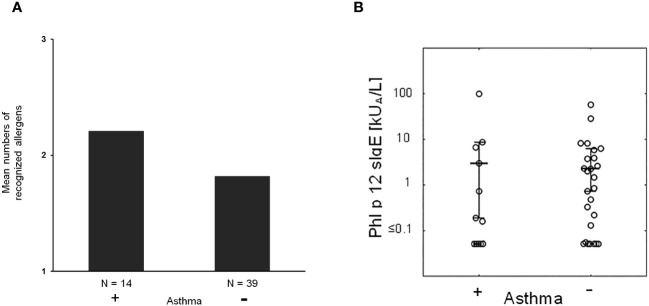
**(A)** Mean numbers of recognized allergens in patients with (+) and without asthma (−). **(B)** Phl p 12-specific IgE in patients with (+) and without (−) asthma.

Next, we studied if severe symptoms, in particular asthma, are associated with the number of recognized allergen molecules. In fact, it seemed that patients with asthma reacted with more allergen molecules than patients without asthma, but there was no statistically significant difference (**Figure 5A**).

Then, we took the possibility into consideration that allergen-specific IgE levels may be associated with asthma. However, there was no apparent association between IgE levels specific for natural *S. kali* allergens and asthma (**Supplementary Table 1**). There was also no difference regarding profilin-specific IgE levels in patients with and without asthma (**Figure 5B**). However, the fact that several patients with monosensitization to profilin (i.e., patient #8, 18, 39, 45, and 46; **Supplementary Table 1**) exhibited allergic symptoms to saltwort pollen confirmed the clinical importance of profilin monosensitization. In fact, each of the aforementioned profilin-monosensitized patients had clinical symptoms to *S. kali* (**Supplementary Table 1**). There was no apparent difference regarding clinical symptoms in oligo- and profilin-monosensitized patients when comparing the whole study population with the profilin-monosensitized patients regarding the type of symptoms (**Table 1**, **Supplementary Table 1**).

**Figure 6:**
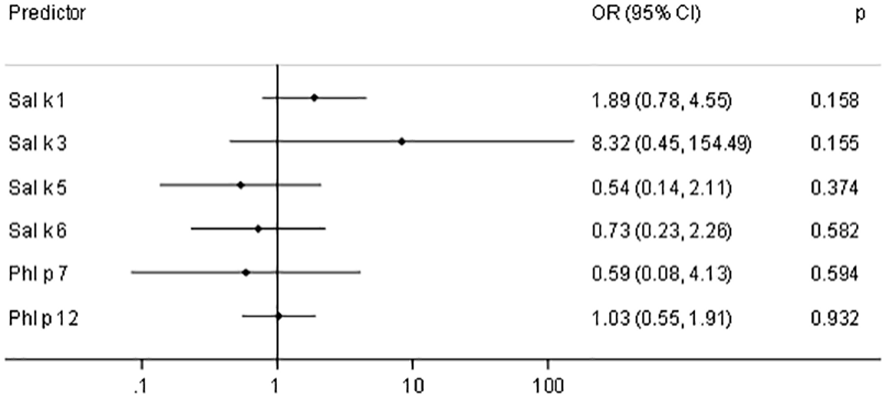
Odds ratios (OR) and 95% confidence intervals (CIs) for a 10-fold increase of IgE (kU_A_/L) with respect to asthma prevalence in saltwort-sensitized patients (*n* = 53).

**Figure 6** shows the odds ratios (ORs) and 95% confidence intervals (CIs) for a 10-fold increase of IgE (kU_A_/L) with respect to asthma prevalence in saltwort-sensitized patients. Here, we noted elevated ORs for Sal k 1 and Sal k 3, the latter of which does not seem to be relevant due to the low number of sensitized patients. However, the asthma prevalence almost doubles if the IgE values measured for rSal k 1 undergo a 10-fold increase (**Figure 6**).

**Figure 7:**
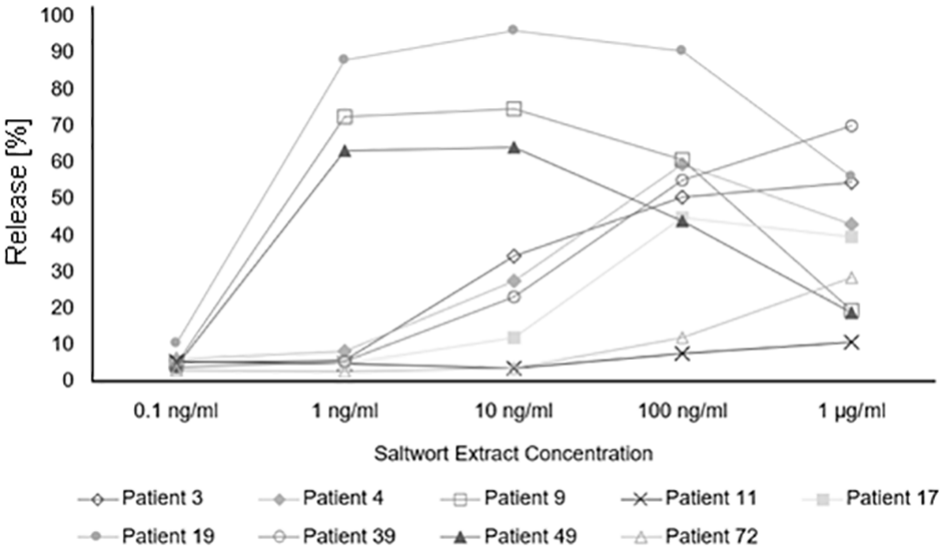
Basophil activation assay. Percentages of total β-hexosaminidase release of patients allergic to *Salsola kali* pollen are shown for different concentrations of the saltwort extract.

Finally, we studied in basophil activation experiments whether the allergenic activity of natural *S. kali* allergens may be associated with allergen-specific IgE levels and number of symptoms. For this purpose, basophils expressing the human high-affinity receptor for IgE were loaded with sera from patients with different levels of allergen-specific IgE and then challenged with allergen extract. Allergen-specific IgE levels in patients with low allergen sensitivity (i.e., patient #39, 3, 4, 17, 72, and 11) ranged between 8.71 and 10.9 kU_A_/L of Sal k-specific IgE whereas those of patients with high sensitivity (i.e., patient #19, 9, and 49) ranged between 8.1 and 27.8 kU_A_/L and thus were higher (**Figure 7**, **Supplementary Table 1**). However, the three patients with high sensitivity exhibited only one to two symptoms whereas two out of the six patients with low sensitivity had three symptoms and the other four patients had one to two symptoms (**Figure 7**, **Supplementary Table 1**). Thus, we have no evidence that the allergenic activity measured in our basophil experiment is associated with severity/number of symptoms.

## 3 Discussion

*S. kali* pollen is an important independent allergen source in many parts of the world, especially in Middle Asia (35). To the best of our knowledge, our study is the first to compare the frequency of IgE reactivity and the corresponding allergen-specific IgE levels of the saltwort allergens Sal k 1, 2, 3, 5, and 6 and the immunological equivalents of Sal k 4 and Sal k 7 from timothy grass pollen, Phl p 12, and Phl p 7, in a population of clinically well-characterized *S. kali*-allergic patients. For the analysis of IgE reactivity and the determination of allergen-specific IgE levels, we used recombinant allergens produced in *E. coli*, to be able to determine IgE antibodies directed only against protein moieties and to avoid IgE reactivity to CCDs. In fact, IgE sensitization to CCDs may originate from sensitization to many *S. kali*-unrelated allergen sources and thus pretend *S. kali*-specific IgE sensitization (31, 36, 37). Furthermore, CCDs are considered to have poor allergenic activity and low clinical relevance (37, 38). *E. coli*-expressed allergens were examined by CD analysis to ensure that the proteins are folded and represent the protein backbone of the different *S. kali* allergens in the best possible way. For the quantification of allergen-specific IgE levels, we used the well-established ImmunoCAP technology. We were thus able to compare IgE levels specific for the individual *S. kali* allergens and their cumulative allergen-specific IgE levels with those specific for natural *S. kali* allergens. For this purpose, each of the recombinant *S. kali* allergens was coupled to streptavidin ImmunoCAPs as previously described (39). Using sera from clinically well-described *S. kali*-exposed patients, we were able to investigate possible relationships between serological results obtained by molecular allergy diagnosis and clinical phenotypes of *S. kali* allergy.

Our study revealed several findings, which can be considered to be important and new for the molecular diagnosis and allergen-specific vaccination approaches for *S. kali* allergy. We confirm that Sal k 1 is a major allergen and specific marker for *S. kali* allergy. However, profilin was identified as allergen accounting for the highest levels of *S. kali*-specific IgE. Importantly, a relevant percentage of saltwort-allergic patients who were negative regarding IgE reactivity for Sal k 1 were detected by profilin (i.e., 37%). Importantly, we expressed and purified the saltwort profilin Sal k 4. Sal k 4 showed an almost identical fold determined earlier for Phl p 12 (32). Sera available from 29 Phl p 12-positive patients showed equivalent IgE binding to Sal k 4 and some patients showed even higher IgE levels to Sal k 4 than to Phl p 12, indicating that Sal k 4 is the genuinely sensitizing allergen in these saltwort-allergic patients. The assumption that Sal k 4 is a clinically important saltwort allergen is supported by the finding that several patients with monosensitization to profilin showed symptoms of respiratory allergy upon saltwort exposure during its flowering season. It is another interesting aspect of our study that IgE levels to Sal k 4 were higher than those to Phl p 12 in several patients. This finding indicates that even highly cross-reactive allergens such as profilin may be useful for component resolved diagnosis to detect the genuinely sensitizing allergen sources. Specifically, higher IgE levels against profilin from one source (e.g., saltwort) as compared to those directed to profilin from another source (e.g., timothy grass) may indicate that the allergen source from which the profilin with the higher IgE binding capacity (i.e., saltwort) is derived may be a genuinely sensitizing allergen source. Such information will be particularly relevant in the absence of IgE sensitization to major allergens.

Profilin has been well studied as cross-reactive allergen in pollen and food derived from different plants. Already shortly after identification of birch pollen profilin as cross-reactive allergen, it was demonstrated that profilin-monosensitized patients had symptoms of respiratory allergy (40). Nevertheless, it was reported afterwards that profilin may eventually not be a clinically relevant allergen (41, 42). In the meantime, several studies have confirmed the role of profilin as a clinically relevant allergen in respiratory allergy and in food allergy (43, 44).

Specifically, it was found that profilin-sensitized patients have a higher risk of plant allergy (45). Accordingly, recombinant plant profilins were found to be comparable regarding IgE reactivity and useful for diagnosis of plant polysensitization (45).

Regarding plant food allergy, it was found that PR10 allergic patients with profilin co-sensitization have more frequent OAS (46) and profilin sensitization was reported to be important for OAS especially in childhood (47). Several other studies including our current study support the role of profilin as a clinically important allergen. For example, it was found that profilin can be a primary airborne sensitizer (48). According to frequencies of IgE recognition, profilin is an important allergen in ragweed pollen allergy (49) and in mugwort pollen allergy (18). For several other allergen sources, profilins were reported to be major allergens such as for eggplant (50), Amaranthus (51), melon (52–54), orange (55), and oilseed rape (56).

We found that by using the combination of Sal k 1 and profilin, more than 81% of patients with saltwort-specific IgE sensitization could be detected, which was close to the percentage achieved by the combination of all recombinant *S. kali* allergens, profilin, and Phl p 7 (i.e., 91%). IgE levels specific for *S. kali* allergen extract in the five patients who were negative for the combination of recombinant allergens were relatively low (i.e., 1.53–5.95 kU_A_/L).

Because of limitations regarding available amounts of sera, we could not test these five patients in the basophil activation test to determine whether they might have been sensitized to allergen components with high allergenic activity, but we found that mainly patients with high levels of saltwort pollen extract-specific IgE levels showed high sensitivity in the basophil activation experiments. The fact that certain patients may be sensitized to saltwort allergens, which have not yet been discovered, is a limitation of our study, but does not affect our major finding that Sal k 4 is an important saltwort allergen.

We could not establish significant correlations between the number and type of symptoms and the molecular IgE sensitization profiles or the basophil sensitivity to allergen extract. Yet, there was a trend that patients with asthma reacted with more allergen molecules than patients without asthma. Furthermore, the asthma prevalence seemed to double if the IgE values measured for rSal k 1 underwent a 10-fold increase, but this was also not statistically significant. Accordingly, other factors may influence the number and severity of symptoms in *S. kali-*allergic patients. These factors may include epithelial barrier disturbances in certain patients and also varying concentrations of the individual allergens in pollen, which may affect the exposure of patients to the individual allergens.

However, our results are highly relevant because they indicate that profilin is an important, if not major allergen in *S. kali* allergy. The clinical role of profilin is underlined by the fact that profilin-monosensitized patients showed respiratory allergic symptoms to saltwort pollen. Therefore, profilin should be taken into consideration when establishing a diagnostic panel of allergen molecules and producing molecular allergy vaccines for the treatment and prevention of saltwort allergy.

## 4 Materials and methods

### 4.1 Characterization of *Salsola kali*-allergic patients

The demographics and clinical characterization of the *S. kali*-allergic patients (*n* = 89) and the one sensitized person without symptoms are summarized in **Supplementary Table 1**. Clinical symptoms related to *S. kali* exposure were recorded by trained allergologists. Each patient was tested for the presence of specific IgE antibodies using *S. kali* extract (w11) with the ImmunoCAP technology (Thermo Fisher, Uppsala, Sweden) to confirm *S. kali* IgE sensitization. A cutoff of 0.1 kU_A_/L was set for a positive reaction. Sera from all patients were also tested for IgE reactivity to multiple allergen components utilizing the ImmunoCAP Immuno solid-phase allergen chip (ISAC) technology.

### 4.2 Expression and purification of allergens

Synthetic genes coding for Sal k 1 [GenBank: AAX11262], Sal k 2 [GenBank: AF449490.2], Sal k 3 [GenBank: ACO34814], Sal k 4.01.01 (**Supplementary Figure 8**), Sal k 5 [GenBank: ADK22842], and Sal k 6 [GenBank: ARS33724] were codon-optimized for *E. coli* expression (ATG:biosynthetics, Merzhausen, Germany). They contain a 3′ DNA coding for a C-terminal hexahistidine tag and were subcloned into the Ndel/EcoRI restriction sites of plasmid pET-17b (ATG:biosynthetics) and expressed in *E. coli* BL-21 Gold (DE3). Freshly transformed cells were grown for 4–6 h at 37°C in a shaking incubator or overnight at room temperature in LB medium supplemented with ampicillin (1 mg/L) until reaching an OD_600 nm_ of approximately 0.6.

Expression of rSal k 1 was induced by the addition of 1 mM isopropyl-beta-D-1-thiogalactopyranoside (IPTG) overnight at room temperature. Cells were harvested by centrifugation in a HITACHI High-Speed Refrigerated Centrifuge CR 22N (Hitachi Koki Co., Ltd., Tokyo, Japan) equipped with R10A5-Rotor at 8 krpm for 10 min (4°C). The pellet was resuspended in 5 mL of lysis buffer (10 mM imidazole, 50mM NaH_2_PO_4_, and 300 mM NaCl, pH 8) prior to the addition of 150 µL protease inhibitor (cOmplete EDTA-free protease inhibitor cocktail, Roche Diagnostics GmbH, Mannheim, Germany; 25× concentrated stock solution, prepared by dissolving one tablet in 2 mL of ddH_2_O). The mixture underwent three cycles of freezing and thawing before being centrifuged at 12 krpm for 30 min. Afterwards, the pellet was discarded and the supernatant was purified via Ni-NTA affinity chromatography (Qiagen, Hilden, Germany) under native conditions, as stated in the Qiagen purification protocol. Pure fractions were pooled, dialyzed into 60 mM Na_2_HPO_4_ buffer (pH 8), and kept as soluble proteins at 4°C until use.

Expression of rSal k 2 was induced by the addition of 1 mM IPTG and the culture was incubated at 18°C for 18 h at 180 rpm. Cells were harvested and the pellet was resuspended in 10 mL of Tris/Triton/imidazole buffer (10 mM Tris, 25 mM imidazole, and 0.1% Triton-X-100, pH 8) prior to the addition of the protease inhibitor (Roche Diagnostics GmbH). The mixture was stirred overnight at 4°C before being centrifuged at 7.8 krpm (Hitachi Koki Co., Ltd.) for 45 min. Afterwards, the pellet was discarded and the supernatant was purified via Ni-NTA affinity chromatography under native conditions following the Qiagen purification protocol. Dialysis of pooled elution fractions was performed against 75 mM NaH_2_PO_4_ (pH 9). The protein was then stored at −20°C.

For rSal k 3, the expression was performed as described for rSal k 2, except that the pellet was resuspended in 5 mL of Tris/Triton lysis buffer, pH 8. Then, 150 µL of protease inhibitor was added and the suspension was stirred for 1 h at room temperature. Subsequently, three cycles of freezing and thawing were performed, and the suspension was centrifuged at 12 krpm for 20 min at 4°C. The pellet was then resuspended in 10 mL of 6 M GuHCl, 100 mM NaH_2_PO_4_, and 10 mM Tris (pH 8). After the addition of 150 µL of protease inhibitor and stirring for 1 h at room temperature, three cycles of freezing and thawing were performed, the mixture was centrifuged, and the supernatant was subjected to nickel affinity chromatography under denaturing conditions (Qiagen). Pooled elution fractions were dialyzed against 75 mM NaH_2_PO_4_ (pH 9) containing 10% glycerol and stored at −20°C.

rSal k 4 was expressed and purified from the soluble *E. coli* fraction by nickel-affinity chromatography as described for Sal k 1 above. Fractions containing purified rSal k 4 were dialyzed against PBS, pH 7.5, and stored in this buffer.

The expression of rSal k 5 was induced by adding 1 mM IPTG into the *E. coli* culture and further incubation at 37°C for 4.5 h under continuous shaking (180 rpm). For rSal k 6, the induction of protein overexpression was initiated by adding 1 mM IPTG to the culture but incubation was continued at 16°C for 48 h under shaking (200 rpm). From the cell harvest onwards, further steps were the same as described for rSal k 3. Pooled elution fractions obtained by nickel affinity chromatography were dialyzed against 75 mM NaH_2_PO_4_ (pH 9) and stored at −20°C.

The purity of the recombinant proteins was analyzed by SDS-PAGE under reducing conditions (**Supplementary Figure 1**). The molecular masses were determined by matrix-assisted laser desorption/ionization time-of-flight mass spectrometry (Axima Confidence, Shimadzu Biotech, Kyoto, Japan) as previously described (57) and subsequent analysis by Bruker Daltonics FlexAnalysis software (**Supplementary Figure 2**).

All recombinant *S. kali* allergens were analyzed by CD spectroscopy [JASCO J-810 spectropolarimeter (Japan Spectroscopic Co., Tokyo, Japan)]. The measurements were performed in a rectangular quartz cuvette with a path length of 0.2 cm for rSal k 1, rSal k 3, and rSal k 5, and 0.1 cm for rSal k 2 and rSal k 6. Spectra were recorded from 195 to 260 nm with a resolution of 0.5 nm at a scan speed of 50 nm/min in triplicate. Then, the spectra underwent a correction by baseline subtraction, the baseline spectra being acquired when the buffers alone were measured. The visualization occurred in the form of mean residue ellipticities calculated at the corresponding wavelength, and secondary structures were determined with the aid of CDSSTR software, which is based on an appropriate reference data set (58, 59) (**Supplementary Figure 3**). For comparison, the structures of the recombinant proteins were predicted using AlphaFold2 (60) on a local installation of the ColabFold server (61). rPhl p 12 was purified and characterized as previously described (32).

### 4.3 Quantification of allergen-specific IgE levels

The ImmunoCAP technology allows the precise quantification of allergen-specific IgE levels (62). For the measurements of IgE levels against the purified recombinant *S. kali* allergens, we used streptavidin ImmunoCAPs available, which provide a matrix binding biotinylated allergen of choice (63). Biotinylation and the verification thereof by ELISA was performed as already described elsewhere (39, 64). To prepare the molecular ImmunoCAP allergens for streptavidin o212 ImmunoCAPs, each recombinant allergen underwent extensive dialysis against 0.1 M NaHCO_3_ and 1 M NaCl at 4°C for at least 8 h. The allergen concentration was determined with the aid of MicroBCA Protein Assay Kit (Thermo Fisher). The streptavidin ImmunoCAPs (o212 Thermo Fisher) were placed in the processing chamber of Phadia® 100 (Phadia AB, Uppsala, Sweden), pre-washed according to the manufacturer’s instructions, and supplemented with the biotinylated recombinant *S. kali* allergens (rSal k 1, 2, 3, 5, or 6) (64). Saltwort extract-specific IgE levels were determined using the commercial ImmunoCAP w11 (Thermo Fisher), rPhl p 7-specific IgE levels were determined using the commercial ImmunoCAP g210, and rPhl p 12-specific IgE levels were determined using the commercial ImmunoCAP g212 (Thermo Fisher). Fluorometrically recorded allergen-specific IgE values above or equal to 0.1 kU_A_/L were considered positive (65).

### 4.4 Comparison of rSal k 4 with rPhl p 12 regarding IgE reactivity

ELISA plates were coated overnight with 2 µg/mL of rSal k 4 and rPhl p 12, respectively, and blocked with 2% BSA-PBSTween blocking buffer. Sera from patients containing Phl p 12-specific IgE and, for control purposes, from a non-allergic subject were diluted 1:10 in PBSTween containing 0.5% BSA and incubated overnight at 4°C. Plates were then washed three times in PBSTween 0.5% BSA and incubated. The horseradish peroxidase-labeled goat anti-human IgE antibodies (KPL, Gaithersburg, MD, USA) diluted 1:2,000 in 0.5% BSA-PBSTween were used for the detection of IgE binding as previously described (66). Results between plates were calibrated using a reference serum. Measurements were carried out as duplicates and results obtained represent averages with a deviation of less than 5%. The results from the non-allergic subject plus 2× SDs were used as a cutoff level.

### 4.5 Statistical analyses

Graphs were generated using Statistica 10.0 (Stat Soft, Tulsa, OK, USA) and GraphPad Prism 8.0.1 (San Diego, CA, USA), and statistical evaluations were performed with Stata 17.0 (StataCorp, College Station, TX, USA). Pairwise comparisons were calculated using the Tukey’s HSD test. The graphical representation for the IgE ELISA data obtained for Sal k 4 and Phl p 12 was performed using GraphPad Prism 6 software (GraphPad Software, La Jolla, CA, USA). The differences between groups were evaluated using an unpaired *t*-test. Correlations of allergen-specific OD levels determined for rSal k 4 and rPhl p 12 by ELISA were assessed by Spearman’s rank correlation coefficient. *p*-values of <0.05 were considered as significant.

### 4.6 *Salsola kali* pollen extract preparation

Pollen from *S. kali* purchased from Stallergenes Greer (Lenoir, NC, USA) had a purity of more than 95%. Two grams of pollen was extracted in 20 mL of phosphate-buffered saline (137 mM NaCl, 2.7 mM KCl, 10 mM Na_2_HPO_4_, and 1.8 mM KH_2_PO_4_, pH 7.4) with 0.1 mM phenylmethylsulfonyl fluoride (1:1,000) and 20 mM ethylenediamine-tetraacetic acid by continuous stirring overnight at 4°C. The supernatant was separated by centrifugation in a HITACHI High-Speed Refrigerated Centrifuge CR 22N (Hitachi Koki Co., Ltd.) equipped with an R20A2-Rotor at 18 krpm for 15 min (4°C) and dialyzed against ddH_2_O at 4°C for 3 days. Afterwards, the extract was centrifuged at the highest speed (20 krpm) for 1 h at 4°C, and its protein content was measured by using the MicroBCA Protein Assay Kit (Thermo Fisher), following the instructions provided by the manufacturer.

### 4.7 Immunoblotting

The detection of profilin in blotted *S. kali* pollen extract was performed by immunoblotting using rabbit anti-Phl p 12 antibodies as previously described (18). Detection of bound Phl p 12-specific rabbit antibodies was performed by the addition of the 1:10,000 rabbit anti-Phl p 12 antiserum and, for control purposes, of an equally diluted normal rabbit serum. Buffers and conditions of the primary detection were as previously described (18). However, the detection of bound rabbit antibodies was performed with alkaline phosphatase-conjugated anti-rabbit IgG from goat diluted 1:2,000 (Dianova, Hamburg, Germany). The color reaction was performed with BCIP/NBT solution and stopped by adding ddH_2_O (67).

The detection of blotted recombinant His-tagged proteins was performed with mouse anti-His antibodies (Dianova, Hamburg, Germany) diluted 1:1,000 in 40 mM sodium phosphate, pH 7.5, 0.5% Tween 20, 0.5% [w/v] BSA, and 0.05% [w/v] NaN_3_ followed by a secondary 1:1,000 alkaline phosphatase-labeled rat anti-mouse IgG_1_ (clone X56, BD Biosciences Pharmingen, San Jose, CA, USA) diluted in the former buffer. Blots were developed in detection buffer [1.65 mg BCIP and 1.65 mg NBT in 10 mL of AP buffer (100 mM Tris, 100 mM NaCl, and 5 mM MgCl_2_)] (67).

IgE immunoblot inhibition experiments demonstrating IgE cross-reactivity between rPhl p 12 and nSal k 4 were performed by pre-incubating serum from a profilin-allergic patient with rPhl p 12 and, for control purposes, with rSal k 5 or BSA (18). Pre-incubated sera were then tested for IgE reactivity to nitrocellulose-blotted *S. kali* pollen extract, and bound IgE antibodies were detected with ^125^I-labeled anti-human IgE antibodies and visualized by autoradiography (18).

### 4.8 Basophil activation test

The culturing of the rat basophil leukemia (RBL) cells expressing human FcϵRI and the subsequent β-hexosaminidase assay procedure were performed as previously described (68–71).

In brief, the cells were cultured in 15 mL of RBL medium [MEM (Gibco)] supplemented with 10% fetal calf serum (Gibco), L-glutamine, penicillin/streptavidin, geneticin and hygromycin B (Life Technologies) at 37°C until confluent. After washing with PBS, the RBL cells were split to reach the density of 1.5 × 10^7^ cells per well. The cells were then seeded (50 µL of cells/well) in a 96-well culture plate (Costar), containing 50 µL of patients’ sera per well, or 50 µL of medium per well for control purposes. The plate was incubated overnight at 37°C. Afterwards, the cells were washed three times with Tyrode’s buffer (134 mM NaCl, 2.6 mM KCl, 1.8 mM CaCl_2_, 1 mM MgCl_2_, 5.6 mM glucose, 417 µM NaH_2_PO_4_, 11.9 mM NaHCO_3_, and 0.1% bovine serum albumin). *S. kali* pollen extract was diluted in Tyrode’s buffer to the corresponding concentrations and added to the wells (100 µL). Negative controls were incubated with Tyrode’s buffer without the extract to calculate spontaneous release. After 1 h at 37°C, an aliquot of 10 µL of 10% Triton X-100 (Sigma) was added to obtain 100% release controls and resuspended. Subsequently, 50 µL of the supernatants was transferred to another 96-well reading plate and supplemented with 50 µL of staining solution (80 µL of 4-methylumbelliferyl-N-acetyl-β-D-glucosaminide in 5 mL of 25 mM citric acid, pH 4.2) each to measure the amount of released β-hexosaminidase. The plate was incubated at room temperature for 1 h. The color development was stopped by the addition of 100 µL of glycine solution (0.2 M glycine and 0.2 M NaCl, pH 10.7) and the absorption was read spectrophotometrically at 465 nm. The results as a mean of three wells were calculated as the percentage of total β-hexosaminidase released after complete cell lysis achieved by the addition of 10% Triton X-100.

## Data availability statement

The original contributions presented in the study are included in the article/**Supplementary Material**. Further inquiries can be directed to the corresponding author.

## Ethics statement

The studies involving humans were approved by ethics committee of the Medical University of Vienna EK1641/2014. The studies were conducted in accordance with the local legislation and institutional requirements. The participants provided their written informed consent to participate in this study.

## Author contributions

LP: Conceptualization, Data curation, Formal Analysis, Investigation, Methodology, Validation, Visualization, Writing – original draft, Writing – review & editing. PG: Investigation, Methodology, Writing – review & editing. DT: Investigation, Methodology, Writing – review & editing. MF-T: Investigation, Methodology, Supervision, Writing – review & editing. VG: Resources, Writing – review & editing. NM: Resources, Writing – review & editing. GD: Resources, Writing – review & editing. NZ: Resources, Writing – review & editing. MI: Resources, Writing – review & editing. BS: Resources, Writing – review & editing. ST: Resources, Writing – review & editing. MT: Methodology, Writing – review & editing. MK: Methodology, Software, Visualization, Writing – review & editing. WK: Methodology, Writing – review & editing. AK: Funding acquisition, Project administration, Writing – review & editing. RV: Conceptualization, Formal Analysis, Funding acquisition, Project administration, Supervision, Validation, Writing – original draft, Writing – review & editing.

## Footnotes

1 WHO/IUIS Allergen Nomenclature Sub-Committee. Salsola kali (2024). http://allergen.org/search.php?allergensource=Salsola+kali [accessed January 29, 2024].

2 1 WHO/IUIS Allergen Nomenclature Sub-Committee. Salsola kali (2024). http://allergen.org/search.php?allergensource=Salsola+kali [accessed January 29, 2024].
