# Supplementary material for: The cytoskeletal protein profilin is an important allergen in saltwort (Salsola kali)
Source: Front Immunol. 2024 Jun 7;15:1379833. doi: 10.3389/fimmu.2024.1379833 (PMC11190152; doi:10.3389/fimmu.2024.1379833)
Supplement: Supplementary file 1 [file DataSheet_1.pdf]

Supplementary Material

1 Supplementary Figures and Tables

1.1 Supplementary Figures

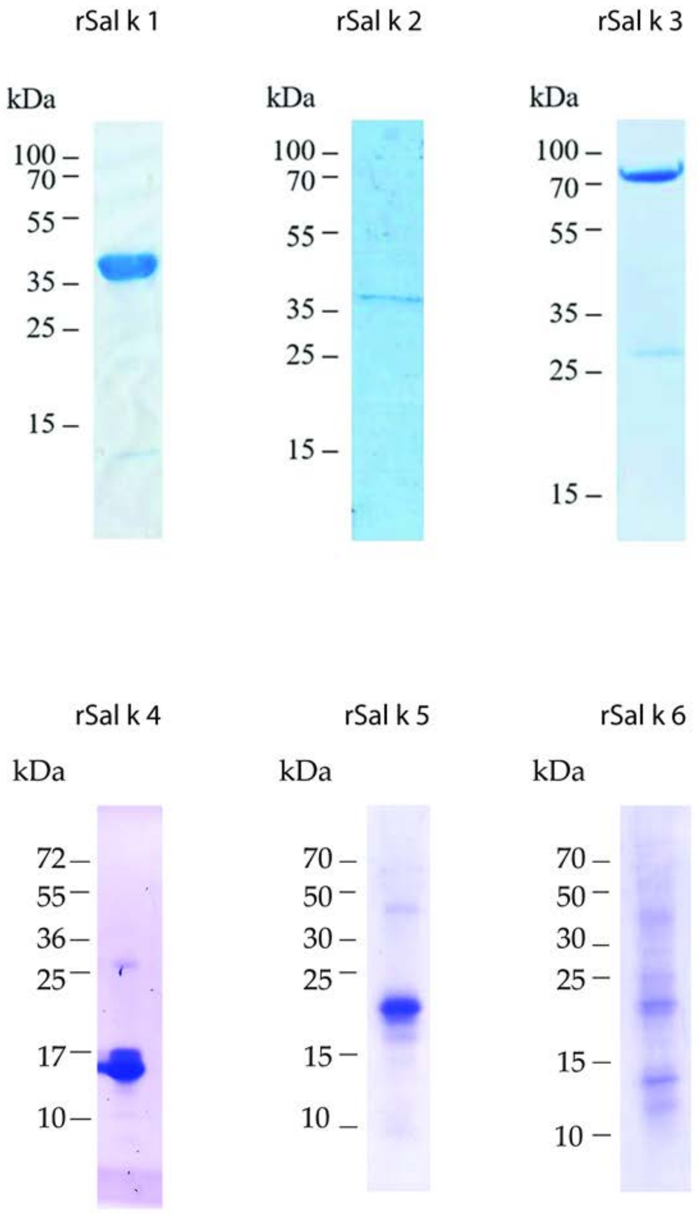

Supplementary Figure 1. Analysis of purified rSal k proteins by SDS-PAGE under reducing conditions with subsequent Coomassie Blue staining. Molecular weight markers (kDa) are shown.

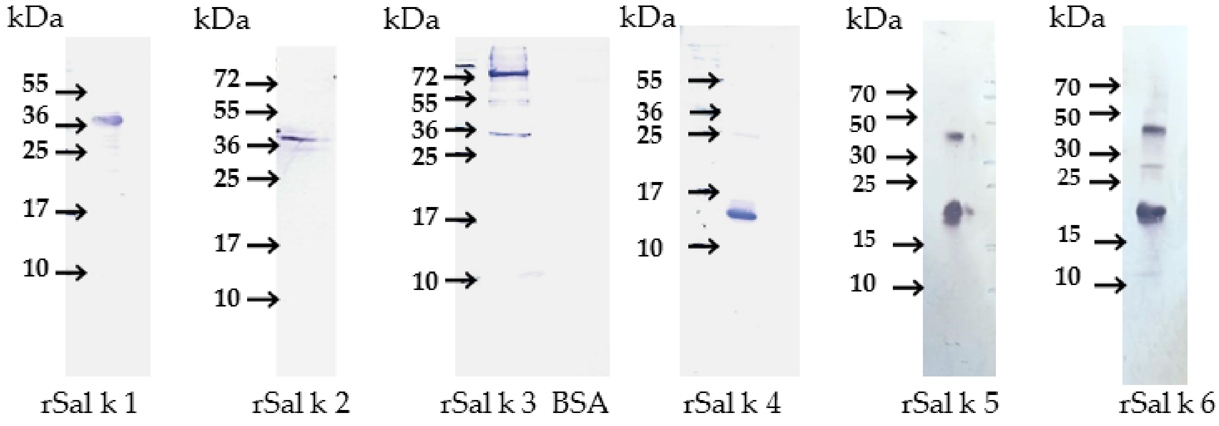

Supplementary Figure 2. Reactivity of blotted rSal k proteins with anti-His tag antibodies. Recombinant His-tagged Sal k allergens (rSal k 1, rSal k 2, rSal k 3, rSal k 4, rSal k 5, rSal k 6) and BSA (non-His-tagged control protein) were separated by SDS-PAGE, blotted onto nitrocellulose and reacted with alkaline phosphatase-conjugated anti-His tag antibodies. Molecular weight markers (kDa) are shown.

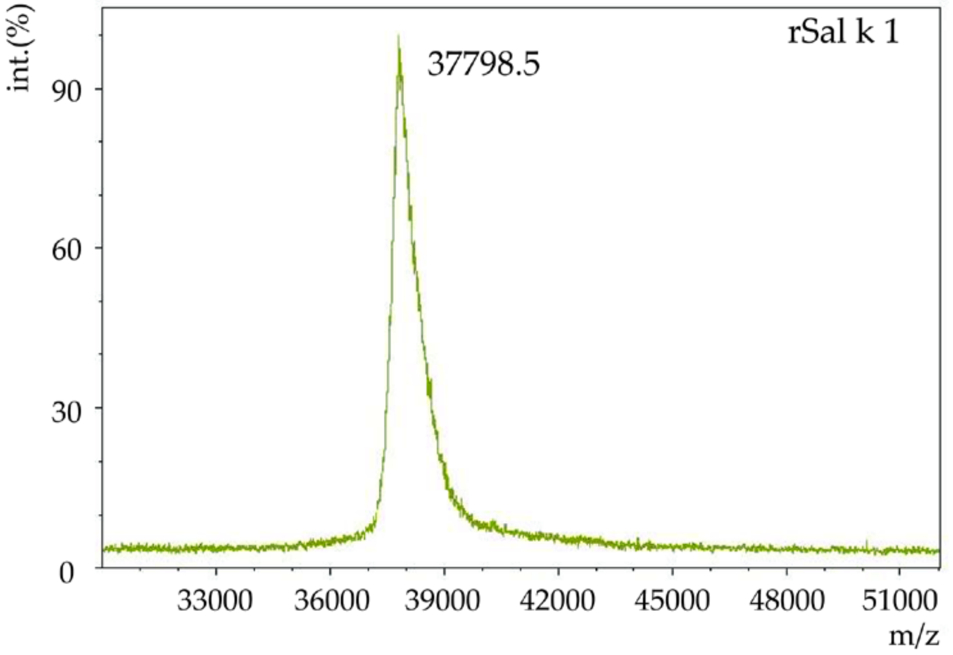
int.(%)
90
60
30
0
37798.5
rSal k 1
33000
36000
39000
42000
45000
48000
51000
m/z

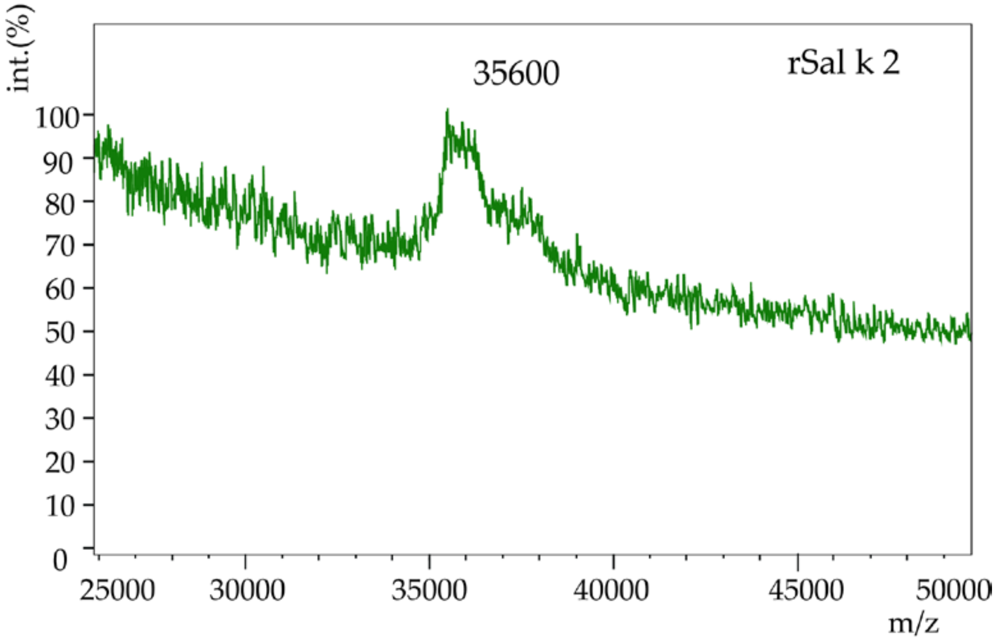
int.(%)
100
90
80
70
60
50
40
30
20
10
0
35600
rSal k 2
25000
30000
35000
40000
45000
50000
m/z

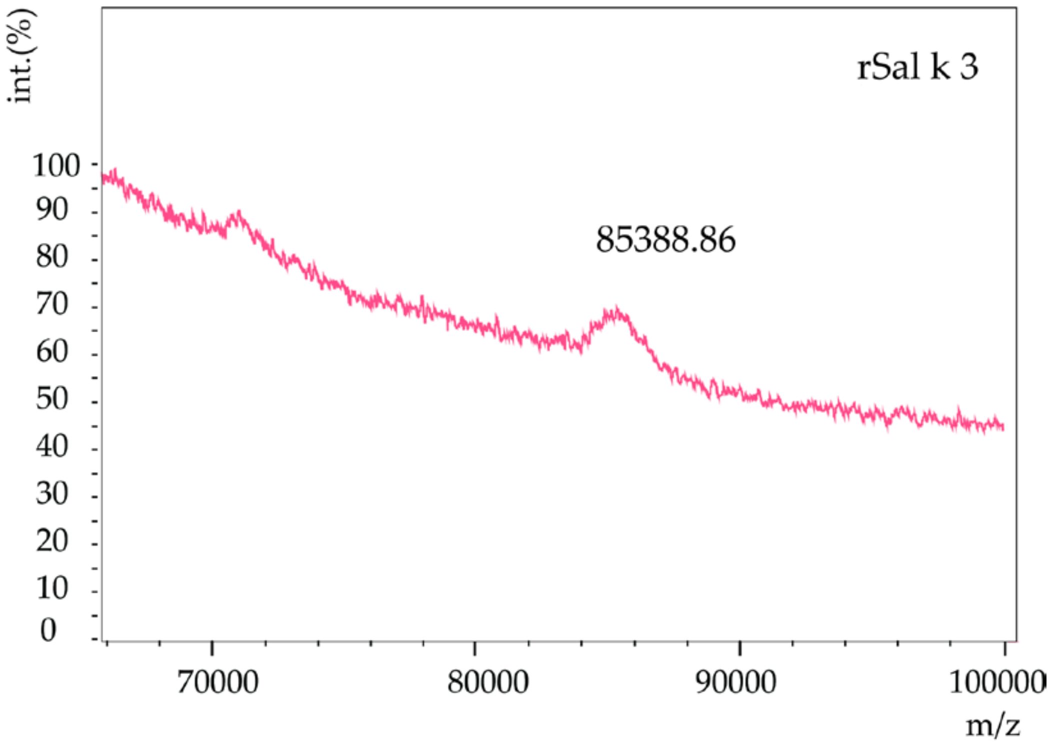
int.(%)
rSal k 3
85388.86
100
90
80
70
60
50
40
30
20
10
0
70000
80000
90000
100000
m/z

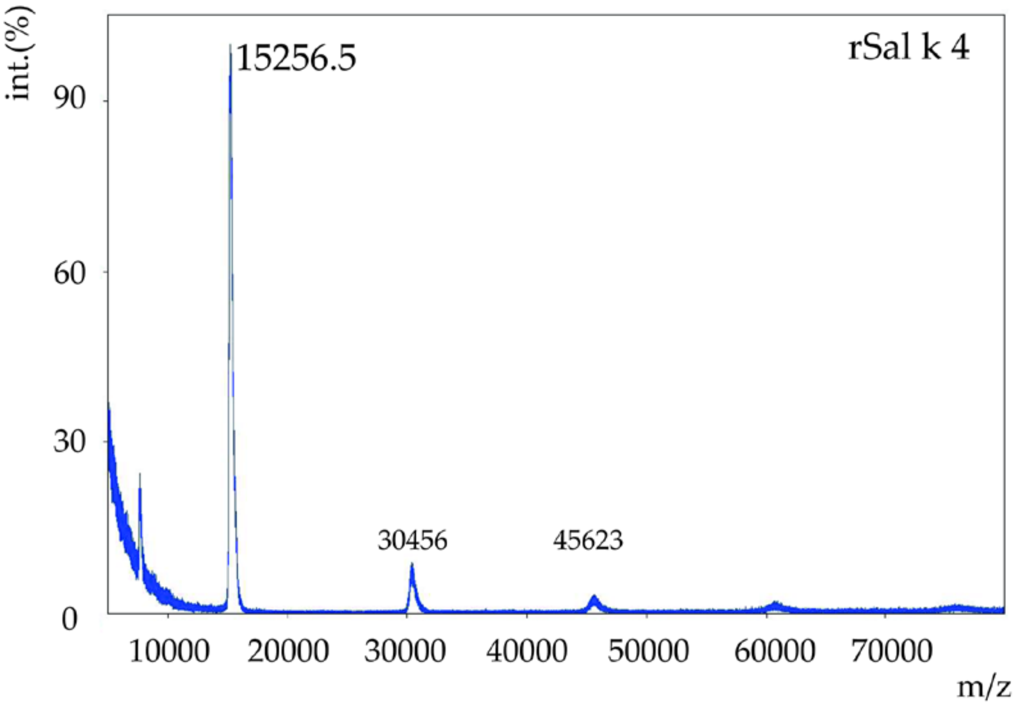
int.(%)
rSal k 4
15256.5
30456
45623
90
60
30
0
10000
20000
30000
40000
50000
60000
70000
m/z

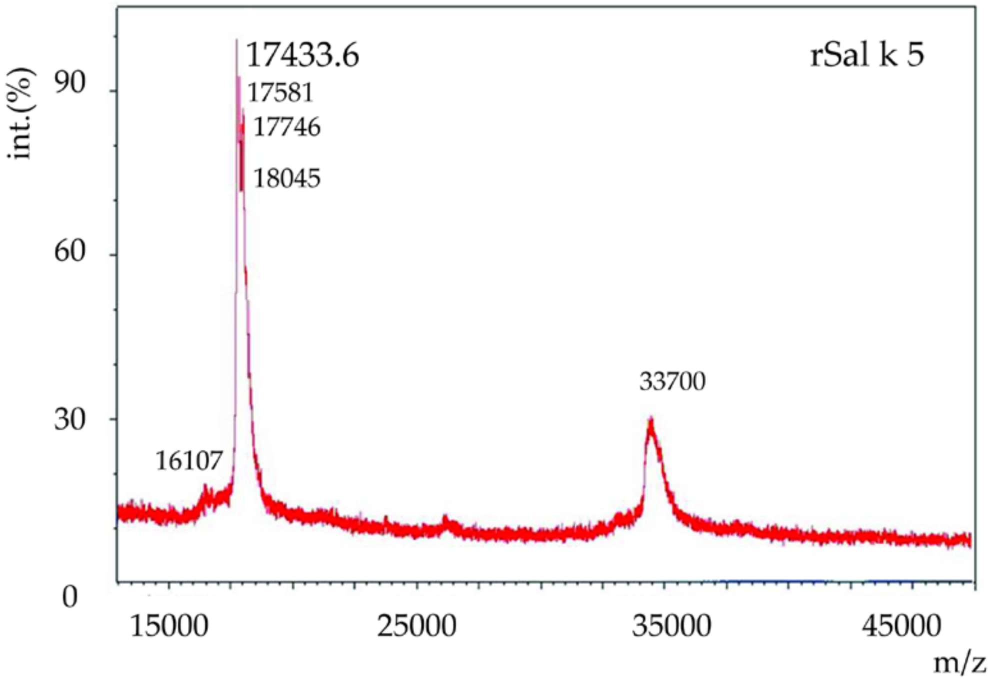
int.(%)
90
60
30
0
17433.6
17581
17746
18045
rSal k 5
33700
16107
15000
25000
35000
45000
m/z

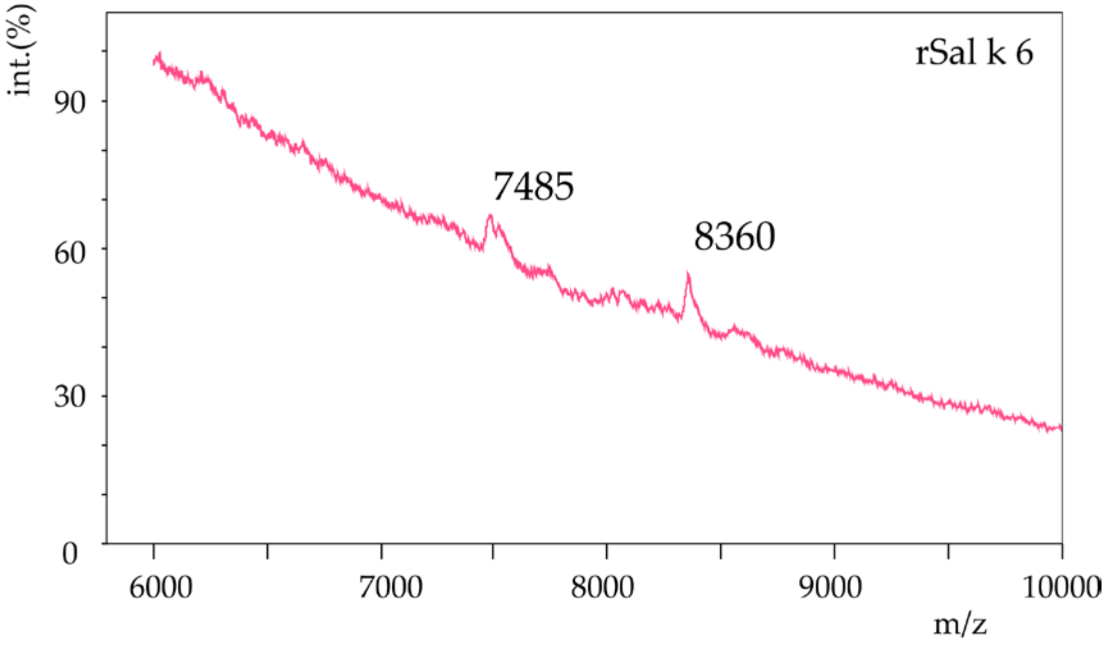
int.(%)
rSal k 6
90
60
30
0
7485
8360
6000
7000
8000
9000
10000
m/z

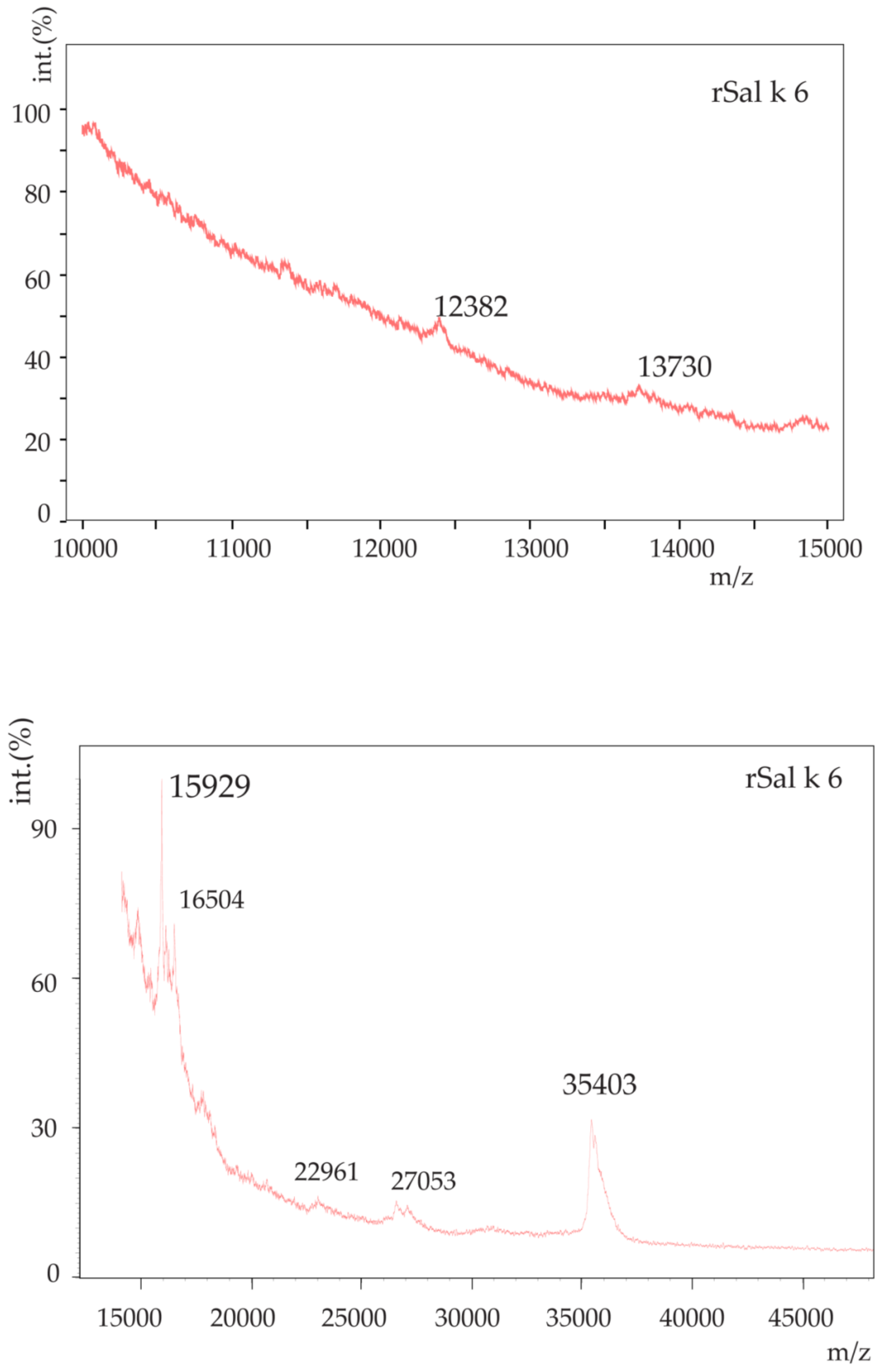

Supplementary Figure 3. Mass spectra of purified recombinant *Salsola kali* allergens (rSal k 1, rSal k 2, rSal k 3, rSal k 4, rSal k 5, rSal k 6). X-axes show the molecular mass (m/Z) which was extended for Sal k 6. Y-axes show the signal intensity as percentage of the most intense signal obtained.

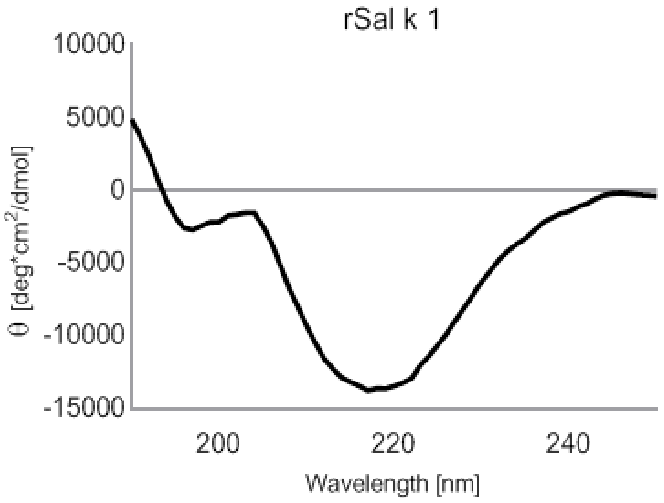
rSal k 1
10000
5000
0
-5000
-10000
-15000
θ [deg*cm2/dmol]
200
220
240
Wavelength [nm]

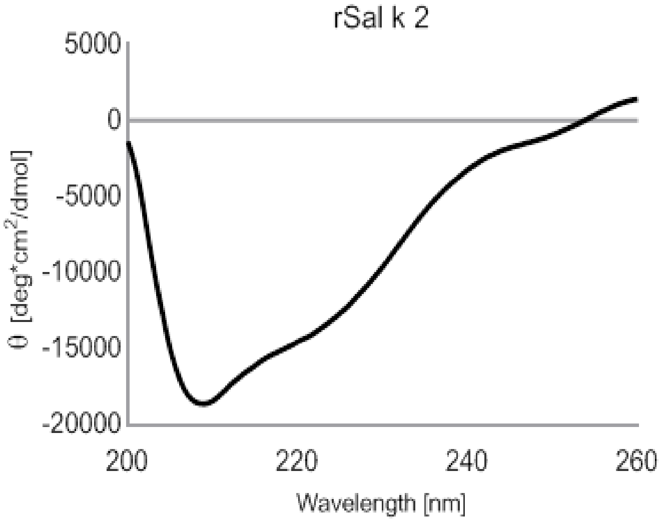
rSal k 2
5000
0
-5000
-10000
-15000
-20000
θ [deg*cm2/dmol]
200
220
240
260
Wavelength [nm]

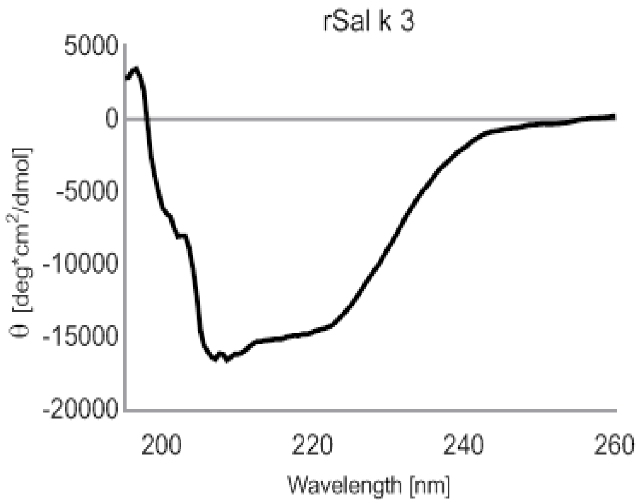
rSal k 3
5000
0
-5000
-10000
-15000
-20000
θ [deg*cm2/dmol]
200
220
240
260
Wavelength [nm]

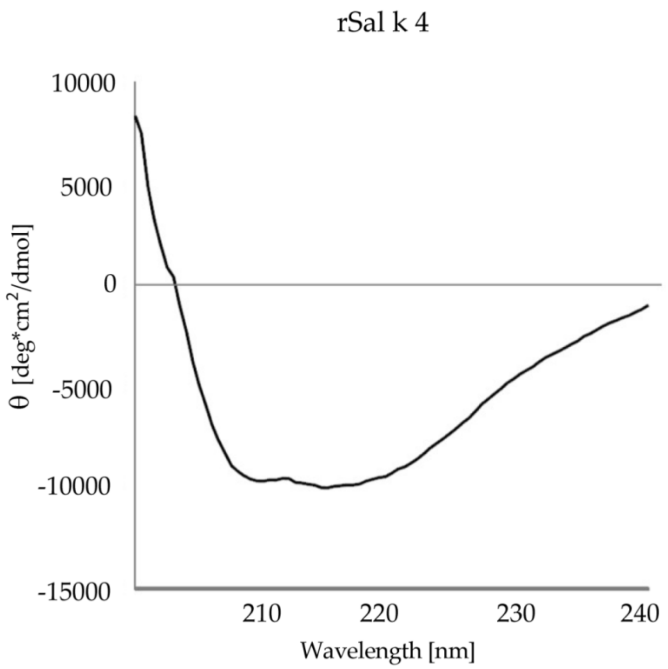
rSal k 4
10000
5000
0
-5000
-10000
-15000
θ [deg*cm2/dmol]
210
220
230
240
Wavelength [nm]

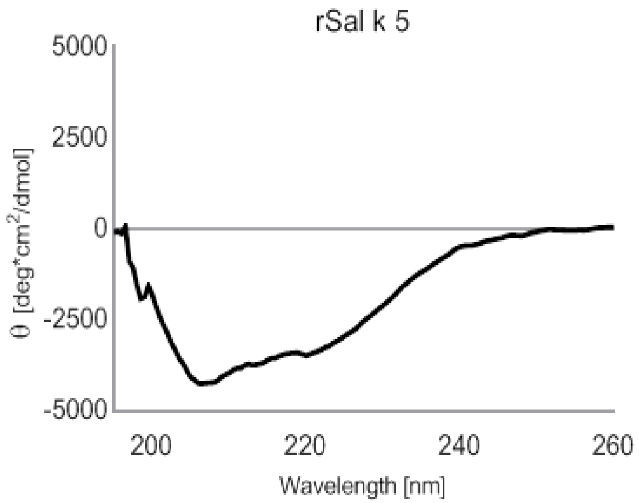
rSal k 5
5000
2500
0
-2500
-5000
θ [deg*cm2/dmol]
200
220
240
260
Wavelength [nm]

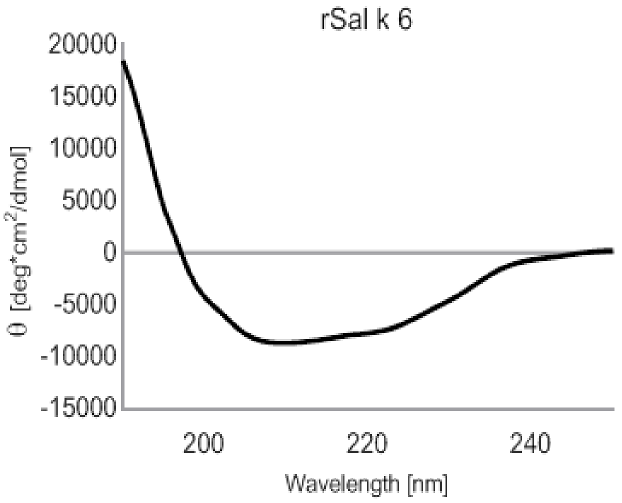

Supplementary Figure 4. Far UV circular dichroism spectra of recombinant *Salsola kali* proteins. The mean residue ellipticities (Θ) (y-axes) are shown at given wavelengths (x-axes) for the individual recombinant allergens rSal k 1, rSal k 2, rSal k 3, rSal k 4, rSal k 5, rSal k 6.

Patient A

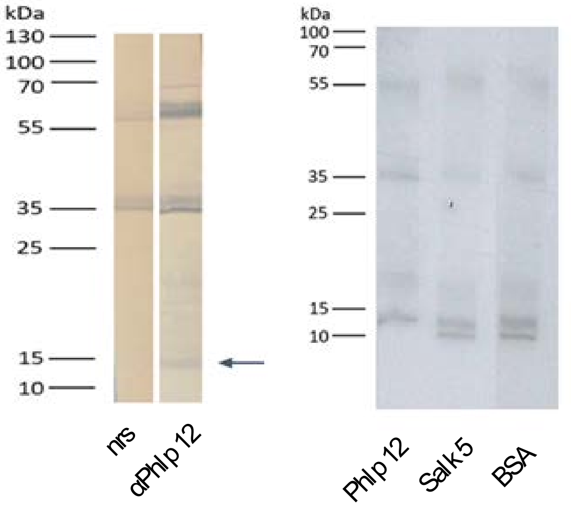

(A) **(B)**

Supplementary Figure 5. (**A**) Detection of *Salsola kali* profilin with rabbit anti-Phl p 12 antibodies in nitrocellulose-blotted *Salsola kali* pollen extract. Lane nrs: normal rabbit serum; lane αPhl p 12: anti-Phl p 12 antiserum. The profilin band is indicated by an arrow. (**B**) Inhibition of IgE binding of a profilin allergic patient to *Salsola kali* pollen extract by rPhl p 12 (lane Phl p 12), rSal k 5 (lane Sal k 5) and BSA (lane BSA). Molecular weight markers in kDa are indicated.

```
Sal k 1.0301    -------------------------QPIPPNPAELESWFQGAVKPVSEQKGLEPSVVQAE    35
Sal k 1.0302    -------------------------...................................    35
Sal k 1.0201    -MEEHVSMLLVG-FVLINIAFTSIAAL...............................T.    58

Sal k 1.0301    SGGVETIEVRQDGSGKFKTISDAVKHVKVGNTKRVIITIGPGEYREKVKIERLHPYITLY    95
Sal k 1.0302    ...................................................G........    95
Sal k 1.0201    ............................................................    118

Sal k 1.0301    GIDPKNRPTITFAGTAAEFGTVDSATLIVESDYFVGANLIVSNSAPRPDGKRKGARASAL    155
Sal k 1.0302    ................................................A...........    155
Sal k 1.0201    ..........................V......S...H...T...............G..    178

Sal k 1.0301    RISGDRAAFYNCKFTGFQDTVCDDKGNHLFKDCYIEGTVDFIFGEARSLYLNTELHVVPG    215
Sal k 1.0302    ........................................L...................    215
Sal k 1.0201    ..............................T...T.........................    238

Sal k 1.0301    DPMAMITAHARKNADGVGGYSFVHCKVTGTGGTALLGRAWFEAARVVFSYCNLSDAVKPE    275
Sal k 1.0302    ............................................................    275
Sal k 1.0201    .........................................D..........L...A...    298

Sal k 1.0301    GWSDNNKPAAQKTIFFGEYKNTGPGAAADKRVPYTKQLTEADAKTFTSLEYIEAAKWLLP    335
Sal k 1.0302    ..........................................................P.    335
Sal k 1.0201    ..............L............P................................    358

Sal k 1.0301    PPKV---        339    Sequence identity %
Sal k 1.0302    ....---        339    98.53%
Sal k 1.0201    ....---        362    94.99%
```

Supplementary Figure 6. Alignment of the amino acid sequences of the Sal k 1.0301 isoform (top) with two other isoforms. Identical amino acids are indicated by dots and gaps by dashes. Amino acids are numbered at the right margin. Sequence identities of Sal k 1.0301 with the other isoforms are shown below. Amino acids with similar properties are colored (light red = acidic hydrophilic, yellow = neutral, light green = basic hydrophilic, light blue = hydrophobic).

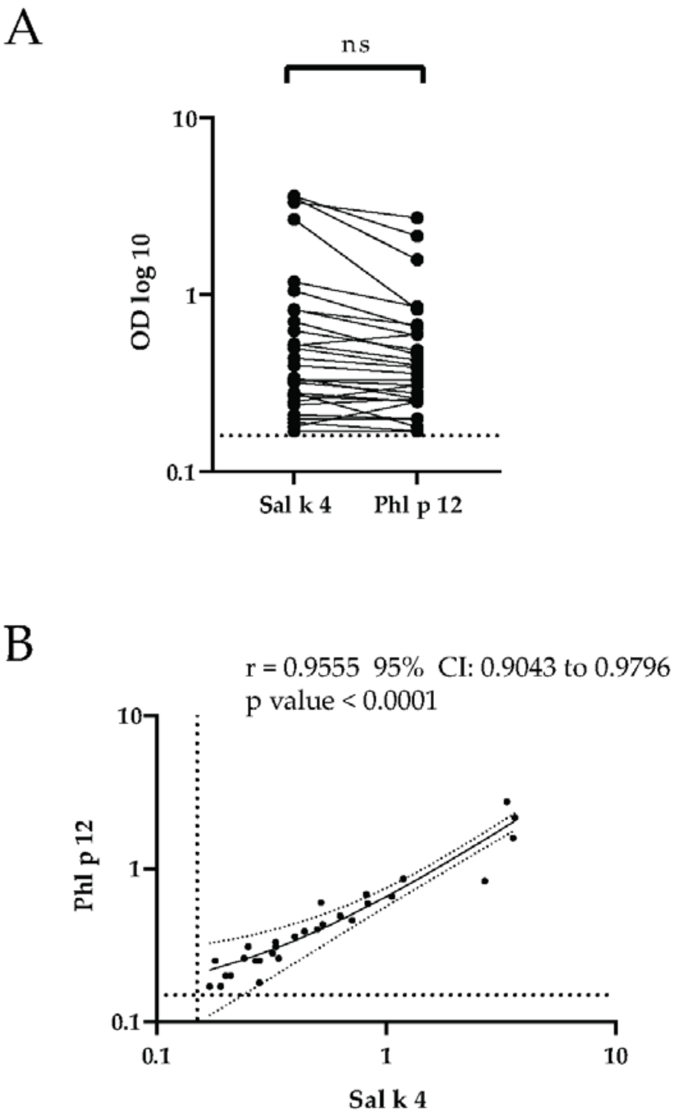

Supplementary Figure 7. (A) IgE reactivity of profilin-sensitized saltwort allergic patients with Sal k 4 and Phl p 12. The y-axis shows the OD values corresponding to the amounts of bound IgE antibodies in a log scale. The horizontal dashed line indicates the cut-off for a positive signal. (B) Correlation of Phl p 12 (y-axis) and Sal k 4 (x-axis) specific IgE levels for the patients shown in (A).

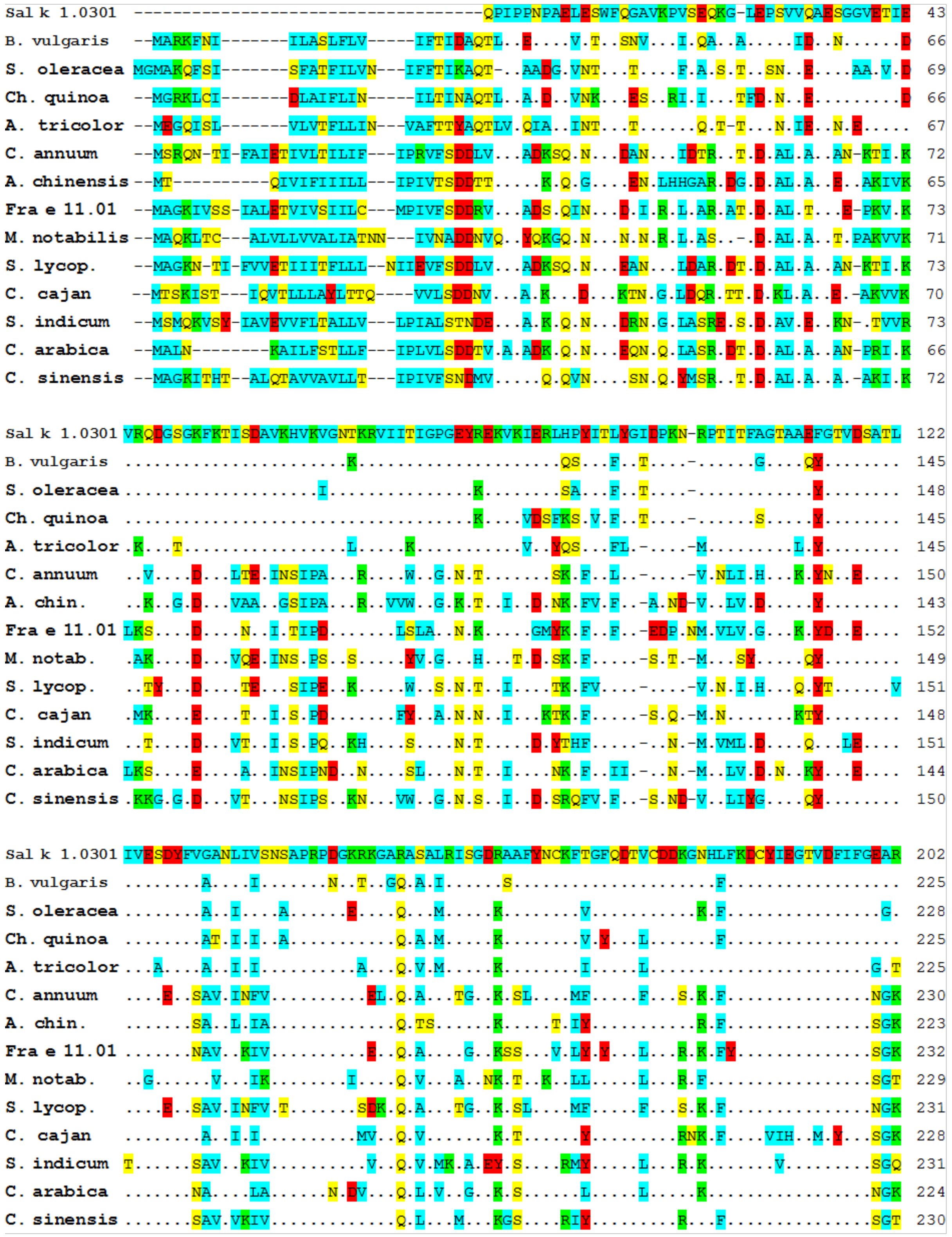

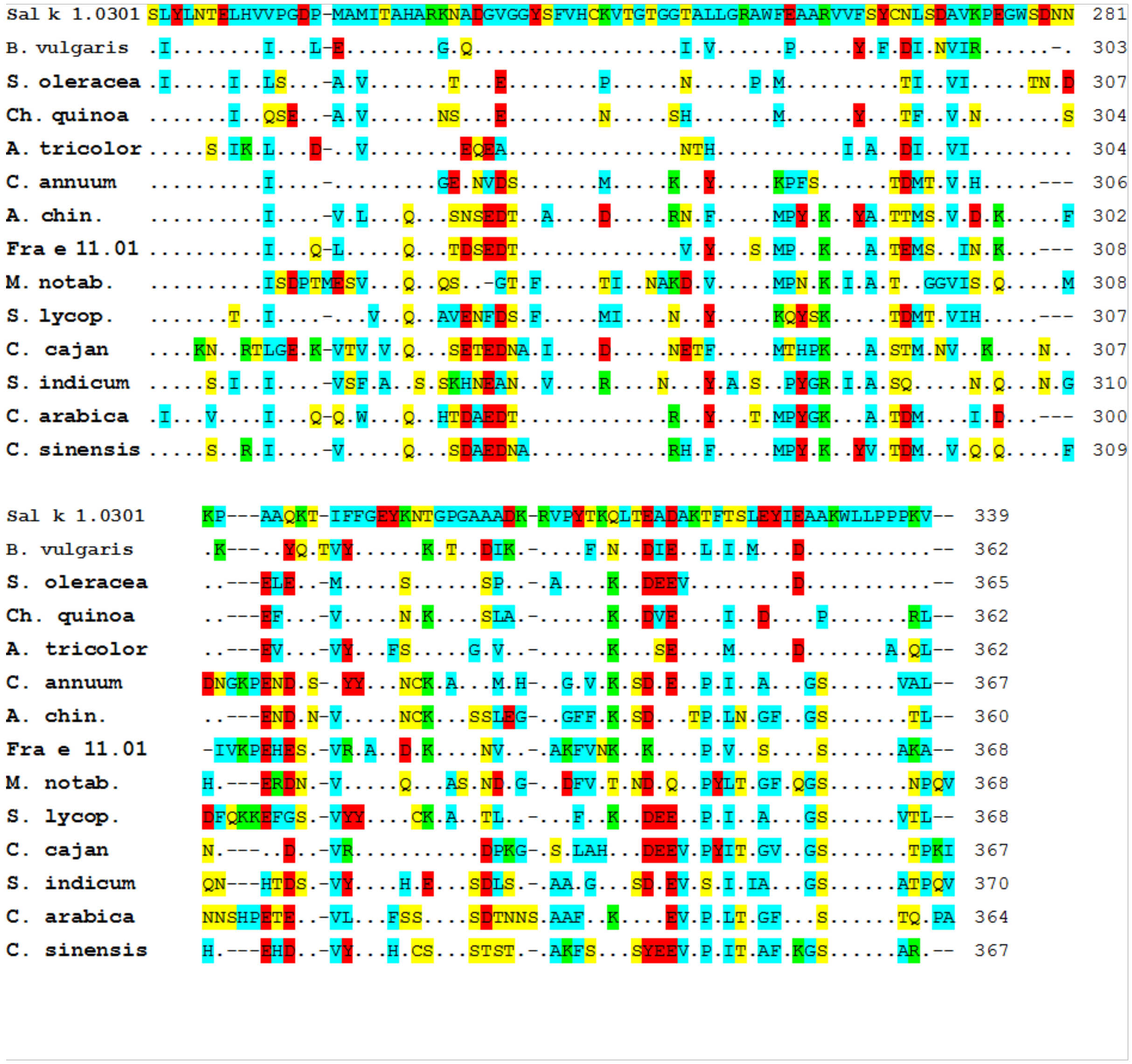

	Sequence identity %
Beta vulgaris subsp. vulgaris (pectinesterase 1)	79%
Spinacia oleracea (pectinesterase 2-like)	79%
Chenopodium quinoa (pectinesterase 1-like)	77%
Amaranthus tricolor (pectinesterase PPME1-like)	77%
Capsicum annuum (pectinesterase 1-like)	63%
Actinidia chinensis var. chinensis (Pectinesterase)	62%
Fraxinus excelsior (Fra e 11.01 allergen)	61%
Morus notabilis (Pectinesterase PPME1)	60%
Solanum lycopersicum (pectinesterase 1-like)	60%
Cajanus cajan (pectinesterase PPME1)	60%
Sesamum indicum (pectinesterase 1-like)	59%
Coffea arabica (pectinesterase 1-like)	59%
Camellia sinensis (pectinesterase 1-like)	58%

Supplementary Figure 8. Alignment of the amino acid sequences of the Sal k 1.0301 isoform (top) with homologous plant proteins (pectin esterases). Identical amino acids are indicated by dots and gaps by dashes. Amino acids are numbered at the right margin. Sequence identities of Sal k 1.0301 with the other pectin esterases are shown below. Amino acids with similar properties are colored (light red = acidic hydrophilic, yellow = neutral, light green = basic hydrophilic, light blue = hydrophobic).

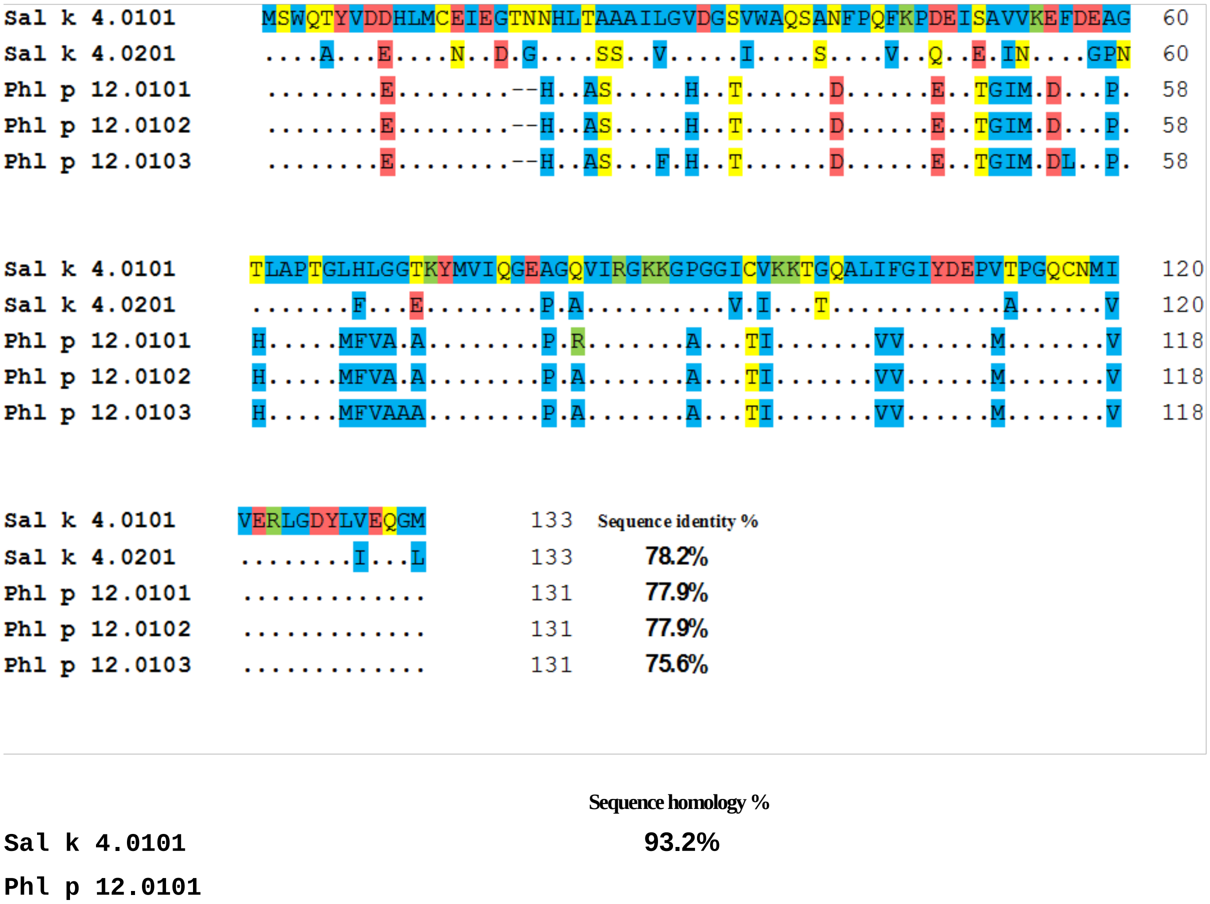

Supplementary Figure 9. Alignment of the amino acid sequences of the Sal k 4.0101 isoform (top) with a second isoforms and three Phl p 12 isoforms. Below the alignment of Sal k 7.0101 with Phl p 7.0101 is shown. Identical amino acids are indicated by dots and gaps by dashes. Amino acids are numbered at the right margin. Sequence identities and homologies are shown displayed. Amino acids with similar properties are colored (light red = acidic hydrophilic, yellow = neutral, light green = basic hydrophilic, light blue = hydrophobic).

1.2 Supplementary Tables

Supplementary Table 1. Demographic, clinical, and serological characterization of the population of saltwort allergic patients. Highlighted are those for whom all components were tested.

1–2 symptoms
3 symptoms
4 symptoms

ID	Sex	Age	Allergic symptoms related to *Salsola kali*				Sal k 1 [kUA/L]	Sal k 2 [kUA/L]	Sal k 3 [kUA/L]	Sal k 5 [kUA/L]	Sal k 6 [kUA/L]	Phl p 7 [kUA/L]	Phl p 12 [kUA/L]	Sum Sal k 1- Phl p 12	Sal k Extract [kUA/L]	HRP [ISU]	Phl p 4 [ISU]	Cup a 1 [ISU]	Cry j 1 [ISU]
			A	R	C	D													
1	m	10	+	+	-	-	n.d.	n.d.	n.d.	-	-	n.d.	n.d.	n.d.	4,49	-	-	-	0,23
2	f	16	-	+	-	-	-	-	-	-	-	-	-	-	1,53	-	-	-	-
3	m	36	-	+	+	+	0,46	-	-	-	-	-	5,87	6,54	8,75	0,56	3,28	0,3	1,43
4	m	9	-	+	+	-	0,15	-	-	-	-	-	-	0,29	9,7	0,11	-	-	0,65
5	f	49	-	+	-	-	-	-	-	-	-	-	-	-	1,79	-	-	-	-
6	f	13	+	+	+	-	1,16	-	n.d.	-	-	-	0,12	n.d.	32,8	-	-	-	-
7	m	34	-	+	+	-	0,11	-	-	-	-	-	-	0,13	3,22	-	-	-	0,15
8	m	40	-	+	+	-	-	-	-	-	-	-	2,31	2,4	4,85	0,24	-	7,13	7,51
9	m	11	-	+	+	-	-	-	-	1,54	3,8	-	-	5,42	18,3	1,18	0,37	3,53	1,58
10	m	7	-	+	-	+	0,48	-	-	-	-	-	0,85	1,38	11,1	0,53	-	-	-
11	m	9	-	+	-	-	0,12	-	-	-	-	-	0,13	0,3	8,71	-	0,09	-	0,15
12	f	12	+	+	-	-	0,56	-	-	-	-	-	-	0,59	0,93	-	0,4	-	0,23
13	f	41	+	+	-	-	0,21	-	n.d.	-	-	-	0,42	n.d.	6,47	0,44	-	-	0,24
14	f	17	+	+	-	-	0,12	0,15	0,11	0,13	0,18	0,1	99,5	100,29	65,1	2,02	0,37	0,09	0,76
15	f	13	-	+	-	-	2,15	-	-	-	-	-	57,4	59,69	84,3	0,68	0,9	0,29	0,62
16	f	32	-	+	+	-	0,39	-	-	4,72	15,5	0,29	2,26	23,19	23,7	0,19	0,53	-	0,28
17	m	62	+	+	+	-	0,23	0,13	0,14	-	0,11	-	0,16	0,91	10,9	0,28	0,19	0,4	1,98
18	m	6	+	+	+	-	-	-	-	-	-	-	3	3,16	3,65	0,17	-	-	0,37
19	f	17	-	+	+	-	0,49	-	-	-	-	-	-	0,65	27,8	-	-	7,36	0,41
20	f	16	+	+	+	+	1,82	-	-	1,99	6,04	-	6,67	16,56	47,2	-	-	6,62	5,67
21	f	19	-	+	+	-	0,15	-	-	-	-	-	3,9	4,11	24,6	0,37	0,12	5,87	2,12
22	f	59	-	+	+	-	n.d.	n.d.	-	0,37	1,63	n.d.	n.d.	n.d.	9,26	n.d.	n.d.	n.d.	n.d.
23	f	12	-	+	-	-	-	-	-	-	-	n.d.	n.d.	n.d.	8,2	n.d.	n.d.	n.d.	n.d.
24	m	45	-	+	+	-	n.d.	n.d.	n.d.	-	-	n.d.	n.d.	n.d.	6,49	n.d.	n.d.	n.d.	n.d.
25	f	13	-	+	+	-	n.d.	-	n.d.	-	-	-	-	n.d.	3,4	-	-	-	-
26	f	9	+	+	+	-	-	-	-	-	-	-	-	-	4,9	-	-	-	-
27	f	8	-	+	-	-	n.d.	n.d.	n.d.	-	-	n.d.	n.d.	n.d.	9,91	n.d.	n.d.	n.d.	n.d.
28	m	46	-	+	-	-	-	-	n.d.	-	-	-	0,32	n.d.	4,15	-	-	2,68	0,99
29	m	32	-	+	+	-	n.d.	n.d.	n.d.	0,1	-	n.d.	n.d.	n.d.	14	-	-	-	0,21
30	m	8	+	+	+	+	0,36	-	-	-	-	-	-	0,44	9,04	n.d.	n.d.	n.d.	n.d.
31	f	28	-	+	-	-	n.d.	n.d.	1,16	8,47	14,4	n.d.	n.d.	n.d.	>100	n.d.	n.d.	n.d.	n.d.
32	m	10	+	+	+	-	n.d.	n.d.	n.d.	-	-	n.d.	n.d.	n.d.	1,7	n.d.	n.d.	n.d.	n.d.
33	m	10	-	+	-	-	n.d.	-	-	0,44	0,96	-	-	n.d.	11,1	-	-	7,31	6,86
34	m	10	-	+	-	+	-	-	-	-	-	-	-	0,18	16,2	-	-	-	-
35	f	52	+	+	+	+	n.d.	n.d.	-	0,28	0,5	n.d.	n.d.	n.d.	3,45	n.d.	n.d.	n.d.	n.d.
36	f	21	+	+	+	-	9,9	0,59	-	-	-	-	8,68	19,24	31,1	-	0,2	-	0,15
37	m	34	-	+	+	-	n.d.	n.d.	n.d.	-	-	n.d.	n.d.	n.d.	8,75	n.d.	n.d.	n.d.	n.d.
38	m	34	+	+	+	-	n.d.	n.d.	n.d.	-	-	n.d.	n.d.	n.d.	4,18	n.d.	n.d.	n.d.	n.d.
39	f	60	-	+	-	-	-	-	-	-	-	-	6,31	6,54	9,87	-	-	-	-
40	m	18	+	+	+	-	0,54	-	1,08	-	-	0,29	0,73	2,69	4,09	-	-	-	-
41	m	51	-	+	-	-	1,23	-	-	-	-	-	0,22	1,54	7,57	0,38	0,09	2,39	0,79
42	f	19	-	+	-	-	0,49	-	-	-	-	-	-	0,56	18,4	-	-	0,77	0,29
43	m	27	-	+	-	-	0,38	-	0,1	1,34	3,1	3,44	0,73	9,1	14,7	0,69	0,11	5,68	2,5
44	m	31	+	+	+	+	n.d.	n.d.	n.d.	-	-	n.d.	n.d.	n.d.	3,09	n.d.	n.d.	n.d.	n.d.
45	m	17	-	+	-	+	-	-	-	-	-	-	8,17	8,31	25,8	-	-	6,17	2,27
46	m	11	-	+	-	-	-	-	-	-	-	-	0,48	0,54	1,1	-	-	-	-
47	m	11	-	+	-	+	n.d.	n.d.	0,18	0,3	0,66	n.d.	n.d.	n.d.	43,6	n.d.	n.d.	n.d.	n.d.
48	f	35	-	+	-	-	0,7	-	-	0,17	0,39	-	-	1,28	4,31	-	-	0,49	0,16
49	f	19	-	+	+	-	0,1	-	-	0,37	0,78	-	-	1,27	8,1	0,59	0,16	0,46	0,27
50	f	56	-	+	+	-	-	-	-	-	-	-	-	0,1	15,2	0,87	0,18	-	-
51	m	27	-	+	-	-	-	n.d.	-	-	-	-	-	n.d.	0,52	-	-	0,19	0,35
52	m	40	-	+	-	-	n.d.	-	-	0,41	0,83	-	-	n.d.	3,51	-	-	1,68	0,98
53	f	29	+	+	+	-	n.d.	n.d.	n.d.	-	-	n.d.	n.d.	n.d.	1,17	n.d.	n.d.	n.d.	n.d.
54	f	18	-	+	-	-	n.d.	-	-	-	-	-	-	n.d.	3,51	-	-	1,94	0,97
55	f	44	+	+	+	-	n.d.	-	-	-	-	-	-	n.d.	0,38	-	0,09	-	-
56	m	29	-	+	-	-	n.d.	-	n.d.	0,1	0,09	-	0,09	n.d.	32,7	7,82	2,25	3,42	6,22
57	f	35	-	+	+	-	n.d.	n.d.	n.d.	-	-	n.d.	n.d.	n.d.	3,02	n.d.	n.d.	n.d.	n.d.
58	m	19	-	+	+	-	n.d.	n.d.	n.d.	-	-	n.d.	n.d.	n.d.	10,1	n.d.	n.d.	n.d.	n.d.
59	m	34	-	+	+	-	n.d.	n.d.	n.d.	0,2	0,29	n.d.	n.d.	n.d.	33,4	n.d.	n.d.	n.d.	n.d.
60	m	5	-	+	+	-	n.d.	n.d.	n.d.	-	-	n.d.	n.d.	n.d.	14,4	n.d.	n.d.	n.d.	n.d.
61	f	57	-	+	-	-	n.d.	-	-	-	-	-	-	n.d.	1,4	8,53	2,55	4,02	7,57
62	f	27	+	+	-	-	n.d.	n.d.	n.d.	-	-	n.d.	n.d.	n.d.	5,43	n.d.	n.d.	n.d.	n.d.
63	m	21	-	+	-	-	-	-	-	-	-	-	-	-	3,47	-	-	1,09	0,77
64	f	38	-	+	-	-	n.d.	-	-	-	-	-	-	n.d.	0,99	-	-	-	-
65	m	13	+	+	+	-	n.d.	-	n.d.	-	-	0,11	0,15	n.d.	22,2	-	-	3,99	3,71
66	f	28	+	+	+	-	-	-	-	-	-	-	-	-	5,95	-	-	-	0,25
67	f	59	-	+	-	-	-	-	-	-	-	-	-	0,21	0,57	9,76	2,23	3,73	8,47
68	f	11	+	+	+	-	n.d.	n.d.	n.d.	0,13	0,09	n.d.	n.d.	n.d.	21,7	n.d.	n.d.	n.d.	n.d.
69	m	15	-	+	+	-	n.d.	-	-	-	-	-	21,7	n.d.	23,1	1,95	0,38	2	4,75
70	m	38	-	+	+	-	-	-	-	-	-	-	-	0,39	11,7	1,12	0,35	0,42	1,27

71	f	7	+	+	-	-	n.d.	-	n.d.	-	-	-	0,3	n.d.	8,65	0,34	-	1,47	2,11
72	m	7	-	+	+	-	-	0,35	-	-	-	-	3,75	4,35	10,1	0,11	-	4,63	1,46
73	f	16	-	+	+	-	0,35	-	0,3	0,32	0,41	0,3	0,33	2,03	26,5	20,98	7,42	17,99	23,09
74	f	15	+	-	-	-	-	0,15	-	-	-	-	-	0,29	2,71	0,35	-	-	0,23
75	m	7	-	+	-	-	0,13	0,52	-	0,11	-	-	1,47	2,49	21	0,96	0,29	0,2	0,93
76	m	60	-	+	-	-	-	-	-	-	-	-	-	0,25	0,77	0,19	-	1,45	0,44
77	m	28	-	+	-	-	-	0,1	-	0,17	0,23	-	8,25	8,9	19,8	0,27	0,25	22,18	2,59
78	f	11	+	+	+	-	2,31	-	-	-	-	-	0,19	2,58	5,52	0,76	0,87	0,09	0,19
79	m	11	-	+	+	-	0,79	-	-	-	-	4,61	28,50	33,94	20,00	1,25	1,96	3,25	1,14
80	m	18	+	+	+	-	2,64	-	-	-	-	-	-	2,73	9,63	-	-	16,16	39,41
81	m	18	+	+	+	-	2,10	-	-	-	-	-	-	2,31	7,11	2,13	0,68	10,54	9,12
82	m	19	-	+	+	-	0,57	-	-	-	-	-	-	0,65	1,46	-	0,23	9,69	12,08
83	f	12	-	+	+	-	3,19	-	-	3,71	0,50	-	-	7,50	19,00	-	0,09	8,55	4,19
84	m	18	-	+	+	-	5,68	-	-	8,51	1,07	-	-	15,32	19,60	3,06	0,82	0,18	0,63
85	m	11	-	-	-	-	1,64	0,35	0,33	0,33	0,31	-	0,10	3,13	10,40	6,82	0,54	1,98	7,77
86	m	13	-	+	+	-	2,00	-	-	-	-	-	-	2,24	4,78	0,09	1,23	-	0,13
87	m	18	-	+	+	+	5,26	-	-	-	-	-	2,01	7,34	11,90	1,02	0,20	16,53	29,81
88	f	17	-	+	+	-	2,62	-	-	-	-	-	-	2,67	7,12	-	-	-	0,18
89	m	16	+	+	+	-	1,32	-	-	-	-	-	-	1,41	3,37	0,26	-	-	0,19
90	m	10	-	+	+	-	0,13	-	-	-	-	-	2,61	3,15	11,50	5,82	4,57	11,60	23,34

Supplementary Table 2. Comparison of OD values corresponding to IgE levels in sera from Phl p 12-positive patients (Table S1) and in a serum from a non-allergic subject specific for Sal k 4 and Phl p 12. Asterisks indicate sera which had been diluted 1:10, the other sera had been diluted 1:5 in the ELISA.

	OD values	
ID	Sal k 4	Phl p 12
P3	0.23	0.22
P8	0.30	0.27
P10	0.23	0.20
P11	0.20	0.18
P13	0.20	0.19
P14*	3.57	1.59
P15*	1.97	1.64
P16	2.14	1.31
P17	0.18	0.19
P18	0.41	0.33
P20*	0.35	0.28
P21	0.52	0.44
P28	0.24	0.19
P36	0.35	0.39
P39*	0.46	0.31
P40	0.14	0.18
P41	0.27	0.24
P43	0.18	0.21
P45*	0.73	0.53
P46	0.23	0.22
P65	0.19	0.14
P69	0.51	0.37
P71	0.14	0.13
P72	1.54	0.50
P73	0.15	0.15
P75	0.32	0.26
P77	0.63	0.40
P87	0.14	0.15
P90	0.13	0.13
Neg. ctrl.	0.12	0.11
SD for Neg. ctrl.	0.0005	0.003
